# Galactose Oxidase Enables Modular Assembly of Conjugates from Native Antibodies with High Drug‐to‐Antibody Ratios**

**DOI:** 10.1002/cssc.202102592

**Published:** 2022-01-20

**Authors:** Antonio Angelastro, Alexey Barkhanskiy, Ashley P. Mattey, Edward G. Pallister, Reynard Spiess, William Goundry, Perdita Barran, Sabine L. Flitsch

**Affiliations:** ^1^ School of Chemistry and Manchester Institute of Biotechnology The University of Manchester 131 Princess Street Manchester M1 7DN; ^2^ The Department of Pharmaceutical Sciences AstraZeneca Silk Road Business Park Macclesfield SK10 2NA UK

**Keywords:** antibodies, antibody–drug conjugates, biocatalysis, glycoengineering, medicinal chemistry

## Abstract

The potential of antibody conjugates with high drug loading in anticancer therapy has recently been highlighted by the approval of Trastuzumab deruxtecan and Sacituzumab govitecan. These biopharmaceutical approaches have spurred interest in bioconjugation strategies with high and defined degrees of drug‐to‐antibody ratio (DAR), in particular on native antibodies. Here, a glycoengineering methodology was developed to generate antibody drug conjugates with DAR of up to eight, by combining highly selective enzymatic galactosylation and oxidation with biorthogonal tandem Knoevenagel–Michael addition chemistry. This four‐step approach offers a selective route to conjugates from native antibodies with high drug loading, and thus illustrates how biocatalysis can be used for the generation of biopharmaceuticals using mild reaction conditions.

## Graphical abstract

**Glycoengineering**: The synthesis of antibody–drug conjugates with high drug loading from native antibodies is of great interest for development of biopharmaceuticals. Here, selective biocatalysis using galactose oxidase and tandem Knoevenagel–Michael addition conjugation chemistry are combined to achieve molecular drug‐to‐antibody ratios of up to eight.

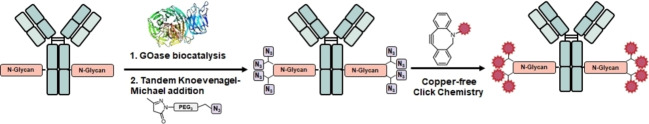

## Introduction

Antibody–drug conjugates (ADCs), the combination between the cancer‐targeting capabilities of monoclonal antibodies (mAbs) and cancer‐killing ability of cytotoxic drugs, are highly regarded in biotherapeutics.^[1]^ Drug‐to‐antibody ratio (DAR), the average number of payloads linked to each antibody, is a crucial attribute in developing an ADC from bench to clinic.^[2]^ While cytotoxicity (and thus potency) is expected to increase linearly with drug loading, the average DAR for most clinically approved ADCs ranges between 2 and 4, which is commonly accepted as the best compromise in obtaining conjugates with favorable therapeutic index.[^2^, ^4^] Nevertheless, the recent approval of Trastuzumab deruxtecan (Enhertu, DAR≈7.7)^[5]^ and Sacituzumab govitecan (Trovedly, DAR≈7.6)^[6]^ prompted a re‐evaluation of high over low‐to‐moderate DAR ADCs.[^4b^, ^7^] The majority of conjugation strategies involve chemical cysteine modification.[^4b^, ^7^] Such approaches can perturb the covalent linkages between the heavy and light chains.^[8]^

With the advancement of site‐specific conjugation techniques,^[9]^ it has become evident that ADCs with precise and homogeneous payload distribution generally show improved pharmacological properties in respect to their mixed‐load counterparts.[^3^, ^10^] However, compared to low‐to‐moderate DAR ADCs,[^3^, ^11^] methodologies for high‐DAR conjugates synthesis are limited;[^10^, ^12^] this issue is remarkably tangible when native antibodies are considered.[^4b^, ^5a^, ^6a^, ^11d^, ^11e^, ^12^]

**Figure 1:**
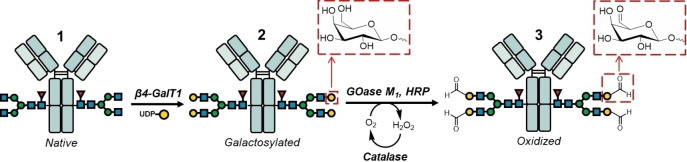
Glycoengineering strategy to introduce four aldehyde groups into Trastuzumab **1** for subsequent site‐specific functionalization. In the first biocatalytic step, β‐1,4‐galactosyltransferase 1 (β4‐GalT1) transfers D‐galactose units from UDP‐Gal to each biantennary *N*‐glycan chain of native **1**. Subsequently, C6‐hydroxy groups of galactose units are oxidized to aldehyde with galactose oxidase (GOase M_1_).

**Figure 2:**
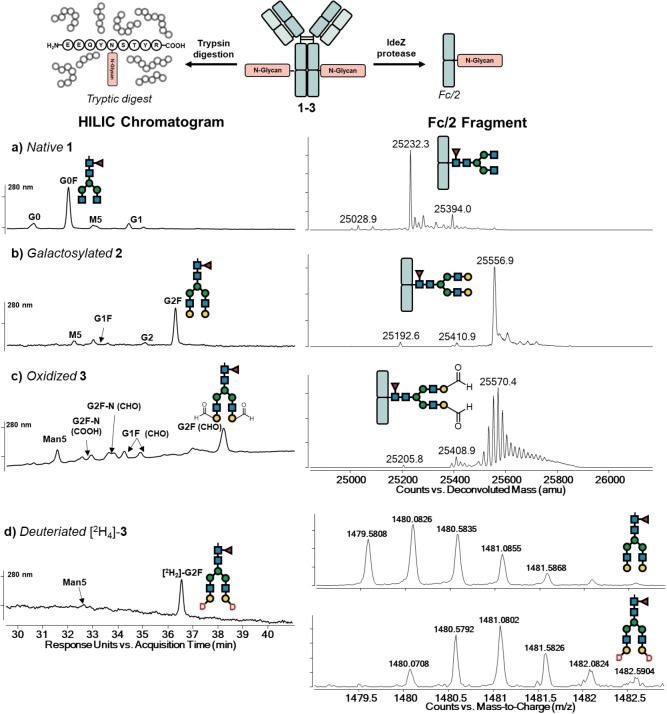
HILIC/MS and Fc/2 analyses of *N*‐glycosylation patterns of (a) native **1**, (b) galactosylated **2**, and (c) oxidized **3** Trastuzumab. (d) HILIC/MS analysis of GOase‐oxidized Trastuzumab [^2^H_4_]‐**3** after reduction of **3** with sodium borodeuteride. See the Supporting Information for details (Figures S2–S24) and experimental procedures.

**Figure 3:**
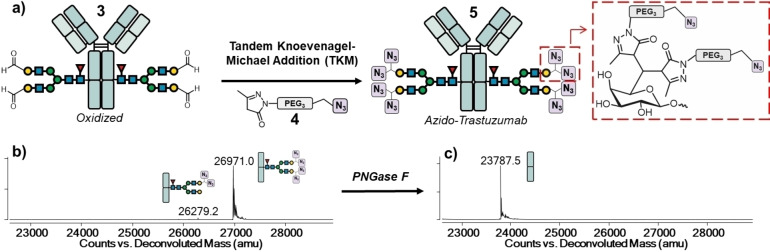
(a) Functionalization of oxidized Trastuzumab **3** with 8 azido groups via TKM. The bifunctional azido‐pyrazolone linker **4** was synthesized in a single step from commercially available starting material (Supporting Information). Fc/2 analysis of the functionalized antibody **5** (b) before and (c) after treatment with PNGase F shows functionalization of the oxidized *N*‐glycan chain with four azido‐pyrazolone linkers being specific.

In this work, we describe an alternative route to the site‐specific production of high‐DAR ADCs by targeting the *N*‐glycan chains of Trastuzumab **1** and using biocatalysis as a mild method for introduction of biorthogonal groups. Key steps involve the enzymatic oxidation of the C6‐hydroxy group of D‐galactose into C6‐aldehyde by galactose oxidase (GOase, Figures 1 and 2),^[13]^ followed by ligation to each aldehyde of two linker‐payload via tandem Knoevenagel–Michael addition (TKM, Figure 3).^[4c]^

## Results and Discussion

During the first biocatalytic step, G0F, the dominant *N*‐glycoform of native Trastuzumab **1**, is galactosylated into G2F **2** using β‐1,4 galactosyltransferase 1 (β4‐GalT1) and uridine diphosphate galactose (UDP‐Gal) as the sugar donor (Figure 1, Figure 2a, b).^[14]^ Subsequently, GOase M_1_, an engineered variant that can be rapidly produced in *E. coli*,^[15]^ was employed to oxidize each galactose unit of the *N*‐glycan into galactoaldehydes **3**.^[13]^ The oxidized Trastuzumab **3** was analyzed after fragmentation by either trypsin to generate glycopeptides or by selective proteolytic digestion using IdeZ protease to generate intact Fc/2 fragments. The fragments were analyzed by two independent and complementary techniques, that is, hydrophilic interaction liquid chromatography/mass spectrometry (HILIC/MS) for the corresponding tryptic digest and reverse phase liquid chromatography/mass spectrometry (RPLC/MS) for the Fc/2 fragments (Figure 2). These were in agreement confirming that G0F sidechains were galactosylated (Figure 2b) and oxidized (Figure 2c) in a highly efficient manner under optimized conditions (Supporting Information).

Although the HILIC chromatogram showed single product peaks, multiple *m*/*z* peaks were observed in the mass spectrum of oxidized Fc fragment of **3**, which we assigned as different hydration states of the aldehydes (Figure 3c). To confirm the identity of the tetra‐aldehyde **3**, oxidized Trastuzumab **3** was subjected to reduction with sodium borodeuteride, and the resulting tryptic digest analyzed by HILIC/MS (Figure 2d). Owing to borodeuteride reduction of aldehydes, conversion of G2F galactoaldehydes into C6‐deuterated galactose ([^2^H_2_]‐G2F) resulted in mass spectra that could easily be assigned to the proposed deuterated product [^2^H_4_]‐**3** and suggested that the previous oxidation was clean.

Having optimized the enzymatic steps to introduce four aldehyde biorthogonal handles on the *N*‐glycan chain of **1**, we next focused on linker‐payload attachment via the TKM reaction (Figure 3a). TKM ligation has been employed in ADC technologies combined to formylglycine‐generating enzyme (FGE),^[16]^ which catalyzes the oxidation of an engineered cysteine residue within a consensus sequence into aldehyde, to produce DAR≈4 ADCs.[^4c^, ^17^] Compared to the FGE/TKM strategy, our GOase mediated *N*‐glycan oxidation would work on native antibodies and not require antibody re‐engineering. In addition, our approach would access high DAR numbers of up to 8 from the tetra‐aldehyde **3**.

As proof‐of‐principle eight azido groups were installed on oxidized Trastuzumab **3** upon reaction with a bifunctional azido‐pyrazolone linker **4** (Figure 3a) to generate azido‐Trastuzumab **5**. Analysis of the Fc/2 fragment of **5** by mass spectrometry clearly showed a major peak at 26971 amu in agreement with the proposed structure and one lower DAR product at 26279 amu as a minor impurity.

The Fc/2 fragment of **5** was further analyzed after deglycosylation with PNGase F (Figure 3c), which shows native mass of 23787 amu and demonstrates that the TKM reaction is highly selective for the *N*‐glycan chain without alterations of the protein backbone. Analysis of deconvoluted MS data of the Fc/2 fragment generated from **5** (Figure S29) indicates G2F decorated with four azido groups being the dominant form (90 %), while *N*‐glycans partially functionalized with two azido moieties represent 2 % of the observed species. Other minor *N*‐glycan species lacking D‐galactose (e. g., Man5), which could not be subjected to GOase oxidation/TKM conjugation, were estimated 8 %. Overall, the approximate average number of azido groups available for conjugation (and thus the theoretical DAR) is 7.3 (see Supporting Information for DAR estimation).

**Figure 4:**
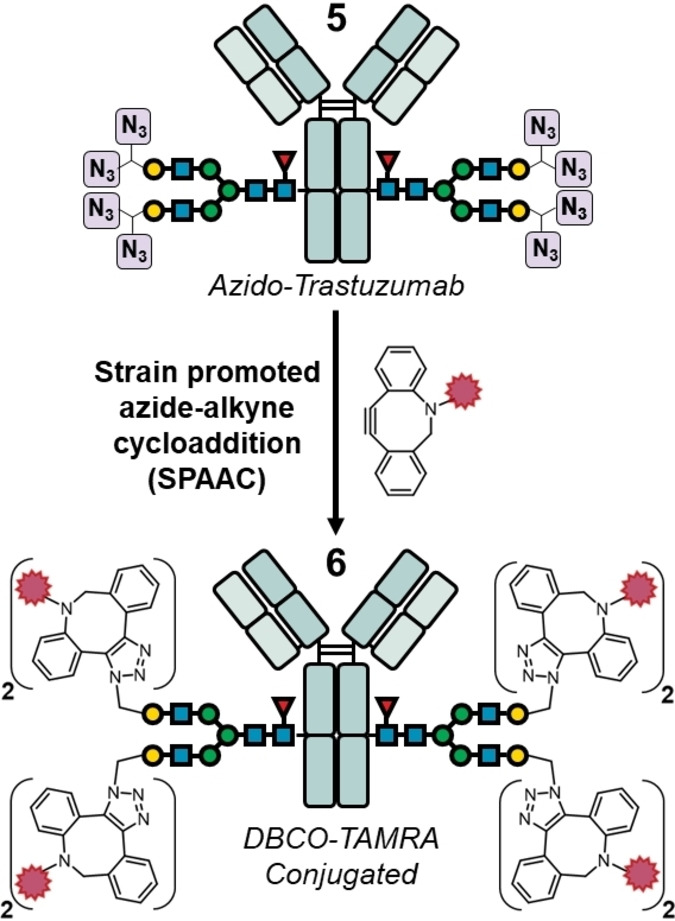
Conjugation of azido‐Trastuzumab **5** to DBCO‐TAMRA by SPAAC.

A mAb functionalized with up to eight azido moieties provides the advantage to rapidly assemble various high‐DAR ADCs via, for example, strain‐promoted azide–alkyne cycloaddition (SPAAC) using different payloads.^[18]^ We exemplified this by reacting azido‐trastuzumab **5** with dibenzylcyclooctyne‐tetramethylrhodamine (DBCO‐TAMRA) to generate the corresponding conjugated antibody **6** (Figure 4 and Supporting Information). Accordingly, RPLC/MS analysis of the Fc/2 fragment generated from **6** shows the *N*‐glycan conjugated to four DBCO‐TAMRA being the dominant species (Figures S28 and S29).

## Conclusion

By combining biocatalysis with tandem Knoevenagel–Michael addition (TKM) chemistry, we have developed a glycoengineering methodology to produce antibody–drug conjugates (ADCs) with a molecular drug‐to‐antibody ratio (DAR) of up to 8 and an average DAR of 7.3. As proof of concept, we have synthesized Trastuzumab functionalized with up to eight azido groups, which could be in turn readily conjugated to different payloads via click chemistry. This enzymatic methodology involving glycosyltransferases and oxidases represents a mild and selective alternative to chemical methods in synthesizing homogeneous high‐DAR ADCs from native antibodies.

## Conflict of interest

The authors declare no conflict of interest.

1

## Supporting information

As a service to our authors and readers, this journal provides supporting information supplied by the authors. Such materials are peer reviewed and may be re‐organized for online delivery, but are not copy‐edited or typeset. Technical support issues arising from supporting information (other than missing files) should be addressed to the authors.

Supporting Information

## Data Availability

The data that support the findings of this study are available in the supplementary material of this article.
