# Supplementary material for: Galactose Oxidase Enables Modular Assembly of Conjugates from Native Antibodies with High Drug‐to‐Antibody Ratios
Source: ChemSusChem. 2022 Jan 20;15(9):e202102592. doi: 10.1002/cssc.202102592 (PMC9303943; doi:10.1002/cssc.202102592)
Supplement: Supplementary file 1 — Supporting Information [file CSSC-15-0-s001.pdf]

ChemSusChem

Supporting Information

Galactose Oxidase Enables Modular Assembly of Conjugates from Native Antibodies with High Drug-to-Antibody Ratios**

Antonio Angelastro, Alexey Barkhanskiy, Ashley P. Mattey, Edward G. Pallister, Reynard Spiess, William Goundry, Perdita Barran, and Sabine L. Flitsch*This publication is part of a collection of invited contributions focusing on “Biocatalysis as Key to Sustainable Industrial Chemistry”. Please visit to view all contributions.© 2021 The Authors. ChemSusChem published by Wiley-VCH GmbH. This is an open access article under the terms of the Creative Commons Attribution License, which permits use, distribution and reproduction in any medium, provided the original work is properly cited.

EXPERIMENTAL PROCEDURES

Materials

Enzymes and antibody. Peroxidase from horseradish (HRP, lyophilized solid, >200 U/mg) and catalase from bovine liver were purchased from Sigma-Aldrich. Recombinant β4-GalT1 from *Homo Sapiens* (ammonium sulfate suspension) and Galactose oxidase M_1 (GOase M_1) fused with a Strep-Tag sequence (lyophilized lysate) were kindly provided by Prozomix Ltd. Sequencing grade trypsin was provided by Promega. Rapid PNGase F was supplied by New England Biolabs. Trastuzumab was provided by AstraZeneca (Gaithersburg, US). Pierce Protein L Chromatography Cartridges (1 mL) were purchased from Thermo Scientific. Strep-Tactin Superflow Plus (1 mL cartridge) was provided by Qiagen.

Chemicals. 1-[Bis(dimethylamino)methylene]-1H-1,2,3-triazolo[4,5-b]pyridinium 3-oxid hexafluorophosphate (HATU) and (3-methyl-5-oxo-4,5-dihydro-1H-pyrazol-1-yl)acetic acid hydrochloride were purchased from Fluorochem Ltd (UK). 11-Azido-3,6,9-trioxaundecan-1-amine was purchased from TCI Chemicals UK Ltd. Sodium borodeuteride (98 atom % D), *d*-desthiobiotin (98%) and phosphate buffered saline (PBS) tablets were purchased from Sigma Aldrich. Dibenzylcyclooctyne-PEG4-5/6-Tetramethylrhodamine (DBCO-TAMRA) was supplied by Jena Bioscience GmbH (Germany). UDP-α-D-galactose disodium salt was provided by Carbosynth Ltd (UK).

GOase M_1 purification and activity assay

GOase M_1 was purified from lyophilized lysate using Strep-Tactin Superflow resin (Qiagen) following instructions provided by the supplier. Briefly, GOase M_1 lyophilized lysate was reconstituted in ~30 mL of 50 mM phosphate, 300 mM NaCl (pH = 8.0), and the resulting solution passed through a 0.45 μm syringe filter before applying it to the resin pre-equilibrated with the same buffer. The resin was then washed three times, and the enzyme was eluted with ~25 mL 5 mM desthiobiotin in 50 mM phosphate, 300 mM NaCl (pH = 8.0). In accordance with previously

established procedures,[1] the purified enzyme was dialyzed against 50 mM sodium phosphate (pH = 7.4) in presence of small quantities of $CuSO_4$, which is needed to ensure GOase M_1 active site being fully loaded with copper.[1] The dialyzed enzyme was then passed through a 0.45 µm syringe filter, buffer exchanged three times with 50 mM sodium phosphate (pH = 7.4) using a centrifugal concentrator (30 kDa cutoff), and concentrated. GOase M_1 activity was measured in accordance to established procedures.[2] Briefly, 10 µL of enzyme was diluted in 90 µl reaction mix (0.23 mg/mL HRP, 0.4 mg/mL ABTS) in 100 mM pH 7.4 sodium phosphate buffer. The reaction was initiated with the addition of 100 µL 50 mM D-galactose in water (final concentration 25 mM). Production of the reduced ABTS was measured using a Tecan infinite 200 plate reader at 420 nm and 30 ºC for 5 min.

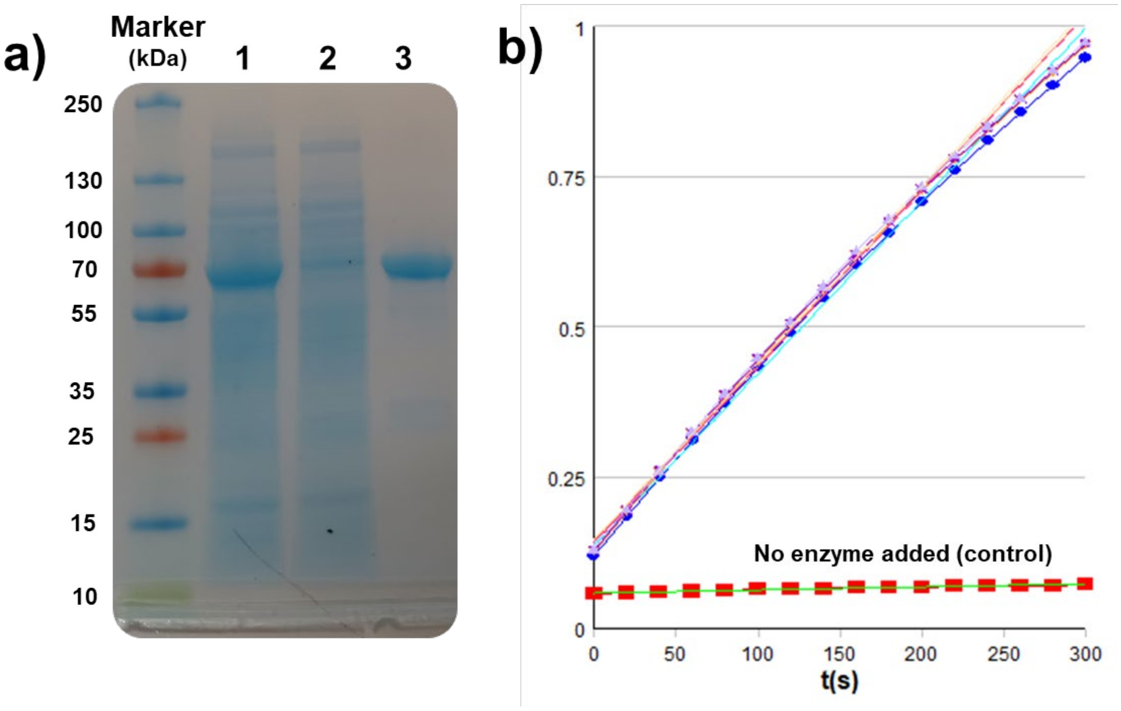

Figure S1. **a**) SDS-PAGE of purified GOase M_1 (70 kDa, lane 3) compared to the reconstituted lysate before (lane 1) and after (lane 2) purification, and **b**) corresponding activity measurements.

Galactosylation of Trastuzumab (1) with β4-GalT1

Trastuzumab **1** (2 mg) in 1 mL 50 mM Tris, 150 mM NaCl, (Tris-buffered saline, TBS), 10 mM $MnCl_2$, pH = 7.6 was incubated with β4Gal-T1 in presence of 20 mM UDP-Gal at 37 °C for 20 h. After incubation, the resulting galactosylated antibody **2** was purified by protein L affinity chromatography. Briefly, the crude antibody was loaded on protein L agarose cartridge (1 mL) pre-equilibrated with TBS, and washed with 10 column volumes of TBS pH = 7.2. The antibody was eluted with 100 mM Gly buffer pH = 2.5; fractions containing the mAb were immediately neutralized with 1 M potassium phosphate dibasic to pH ≈ 7.4 and pooled together. The purified antibody was buffer exchanged in 50 mM sodium phosphate (pH = 7.4) *via* ultrafiltration using a centrifugal concentrator (10 kDa cutoff), and concentrated.

Oxidation of galactosylated Trastuzumab (2) with GOase-M_1

Galactosylated Trastuzumab **2** (1.4 mg, *vide supra*) was incubated for 24 h with GOase M_1 (1.7 mg/mL, 240U/mg) in presence of Horseradish Peroxidase (HRP) and Catalase in 15 mL 50 mM sodium phosphate buffer pH = 7.4. The resulting oxidized antibody was purified by protein L affinity chromatography as for galactosylated Trastuzumab **2** (*vide supra*) following the procedure recommended by the supplier. The purified antibody, oxidized Trastuzumab **3**, was buffer exchanged in Phosphate Buffered Saline (PBS, 10 mM phosphate, 154 mM NaCl, pH = 7.4) *via* ultrafiltration using a centrifugal concentrator (10 kDa cutoff), and concentrated.

Synthesis of azido-Trastuzumab (5)

Oxidized Trastuzumab (**3**, 171 µL, 2.9 mg/mL, ~0.5 mg) prepared as described above, was incubated with 1 mM of pyrazolone-azido linker (**4**, 1 µL of linker stock solution in DMSO 180 mM added) in PBS buffer for 20 h. Excess of linker was removed *via* buffer exchange with PBS using a centrifugal concentrator (10 kDa cutoff).

Conjugation of azido-Trastuzumab to DBCO-TAMRA *via* Strain-promoted alkyne-azide cycloaddition (SPAAC)

Azido-Trastuzumab **5**, prepared as described above (99 µL, 2.9 mg/mL, ~0.3 mg), in PBS buffer was incubated with 100 µM DBCO-TAMRA (1 µL of stock solution in DMSO 10.7 mM added) for 12 h. Excess of DBCO-TAMRA was removed *via* buffer exchange with PBS using a centrifugal concentrator (10 kDa cutoff).

Antibody N-glycan analysis *via* HILIC/MS of the corresponding tryptic digest

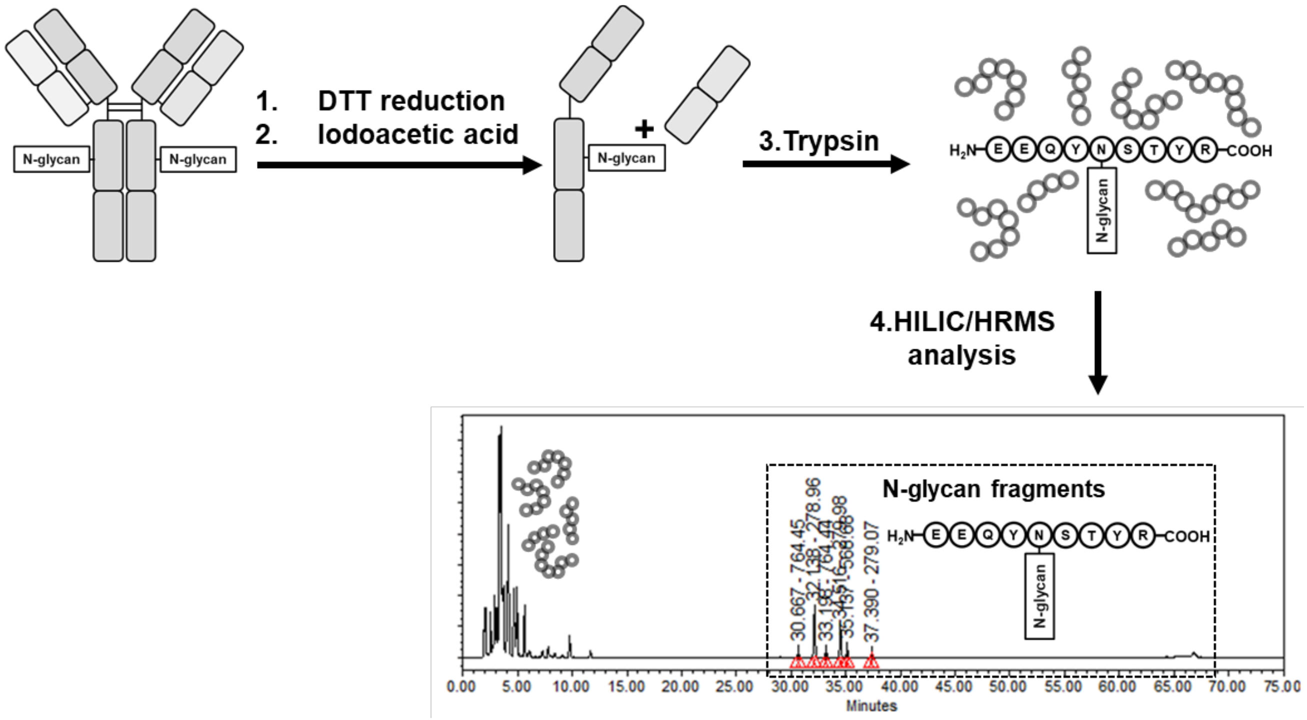

Figure S2. Schematic representation of the general protocol for HILIC/MS analysis of Trastuzumab N-glycosylation pattern.

Preparation of the antibody tryptic digest for HILIC/MS analysis. 100 µL of antibody sample (50 - 100 µg) were combined with 100 µL of 8 M urea (final concentration 4 M), followed by the addition of 30 µL of 10 mM DTT. The mixture was incubated at 56 °C for 30 min, put on ice for 2 min, and most of buffer removed by ultrafiltration using a centrifugal concentrator (10 kDa cutoff). The reduced antibody was exchanged with 100 mM Tris buffer by ultrafiltration (pH = 8.0), to be then treated with 50 µL of iodoacetic acid 15 mM (final volume ~200 µL) for 30 min protected from light.

Excess of iodoacetic acid was removed by ultrafiltration for 10 min, followed by buffer exchange in 50 mM ammonium bicarbonate (pH = 7.8). The antibody in ~50 µL volume was treated with 5 µg of sequencing grade trypsin (5 µL of 20 µg/µL stock solution in 50 mM ammonium bicarbonate buffer) overnight at 37 °C. After overnight incubation, tryptic digest of the antibody was subjected to ultrafiltration, and the filtrate containing tryptic fragments was placed inside a 1.5 mL tube and concentrated under vacuum.

*Reduction of oxidized Trastuzumab **3** with sodium borodeuteride and subsequent digestion with Trypsin for HILIC/MS analysis.* 100 µL of oxidized Trastuzumab **3** (100 µg) in PBS buffer pH = 7.4 were combined with 100 µL of 8 M urea (final concentration 4 M), followed by the addition of 20 µL of sodium borodeuteride 1 M (final concentration 0.1M). The resulting solution was incubated at r.t. for 30 min, followed by removal of borodeuteride by buffer exchange with PBS three times using a centrifugal concentrator (10 kDa cutoff). As borodeuteride also mediated reduction of the protein disulfide bonds,[3] the resulting reduced antibody was exchanged with 100 mM Tris buffer by ultrafiltration (pH = 8.0), to be then treated with 50 µL of 15 mM iodoacetic acid (final volume ~200 µL) for 30 min protected from light. Excess of iodoacetic acid was removed by ultrafiltration for 10 min, followed by buffer exchange in 50 mM ammonium bicarbonate (pH = 7.8). The antibody in ~50 µL volume was treated with 5 µg of sequencing grade trypsin (5 µL of 20 µg/µL stock solution in 50 mM ammonium bicarbonate buffer) overnight at 37 °C. After overnight incubation, tryptic digest of the antibody was subjected to ultrafiltration, and the filtrate containing tryptic fragments was placed inside a 1.5 mL tube and concentrated under vacuum.

Hydrophilic interaction chromatography / mass spectrometry (HILIC/MS) of the antibody tryptic digest. Data was acquired using an Agilent 6520 Ion Mobility LC/Q-TOF system in positive mode (reference mass 922.00979800) equipped with a LC Agilent 1290 Infinity system and Waters ACQUITY UPLC glycoprotein BEH Amide (300 Å, 1.7 µm) column. Column flow was set at 0.2 mL/min. Tryptic digest of the antibody sample (prepared as described above) was dissolved in 20 µL of a solution composed of 80% acetonitrile in water with 0.1% TFA. Injection volume was 5 µL. N-

glycopeptide fragments were separated through a linear gradient of 80% to 50% acetonitrile in 60 min (Table S1). Column temperature was 45 °C. DAD acquisition was set on 215.0, 280.0 and 260.0 nm.

Table S1. Gradient parameters for HILIC separation.

Time (min)	Solvent A (Water 0.1% TFA)	Solvent B (Acetonitrile 0.1% TFA)
**1.00**	20%	80%
**60.00**	50%	50%
**61.00**	80%	20%
**63.00**	80%	20%
**64.00**	20%	80%
**75.00**	20%	80%

Instrument parameters for acquisition were 325 °C gas temp., 8 L/min gas flow, 40 psig nebulizer, 450 °C sheath gas temp., 12 °C sheath gas flow. Scan source parameters were 3500 VCap, 1800 V noozle voltage, 300 fragmentor, 30 skimmer, 750 octopoleRFPeak.

Raw HILIC/MS data was processed using MassHunter Workstation Software Version B.08.00 (Agilent Technologies, Inc. 2016). For each HILIC/MS chromatogram, N-glycoforms were assigned by extracting $[M+H]^{+}$, $[M+2H]^{+2}$, $[M+3H]^{3+}$ predicted ion for each known glycoform (Table S1) followed by manual inspection of the corresponding MS spectrum of each peak from the corresponding EIC (Figures S2-S19).

Table S2. Molecular formulas and corresponding theoretical m/z of possible N-glycopeptides generated from mAb trypsin digestion.

Tryptic N-glycopeptide	Molecular Formula	Theoritical m/z<br>$[M+H]^{+}$, $[M^{+2}H]^{2+}$, $[M+3H]^{3+}$
**Man5**	C96 H148 N16 O55	2405.9349, 1203.4711, 802.6498
**G0-N**	C92 H141 N17 O50	2284.9086, 1142.9579, 762.3077
**G0F-N**	C98 H151 N17 O54	2430.9665, 1215.9869, 810.9937
**G0**	C100 H154 N18 O55	2487.988, 1244.4976, 830.0008
**G1**	C106 H164 N18 O60	2650.0408, 1325.524, 884.0184
**G1 (CHO)**	C106 H162 N18 O60	2648.0251, 1324.5162, 883.3466
**G1 (COOH)**	C106 H162 N18 O61	2664.0201, 1332.5137, 888.6782
**G0F**	C106 H164 N18 O59	2634.0459, 1317.5266, 878.6868
**G1F**	C112 H174 N18 O64	2796.0987, 1398.553, 932.7044
**G1F (CHO)**	C112 H172 N18 O64	2794.083, 1397.5452, 932.0325
**G1F (COOH)**	C112 H172 N18 O65	2810.078, 1405.5426, 937.3642
**G1F-N**	C104 H161 N17 O59	2593.0193, 1297.0133, 865.0113
**G1F-N (CHO)**	C104 H159 N17 O59	2591.0037, 1296.0055, 864.3394
**G1F-N (COOH)**	C104 H159 N17 O60	2606.9986, 1304.0029, 869.671
**G2**	C112 H174 N18 O65	2812.0936, 1406.5504, 938.0361

**G2 (CHO)**	C112 H172 N18 O65	2810.078, 1405.5426, 937.3642
**G2 (2CHO)**	C112 H170 N18 O65	2808.0623, 1404.5348, 936.6923
**G2 (CHO, COOH)**	C112 H170 N18 O66	2824.0572, 1412.5323, 942.0239
**G2 (COOH)**	C112 H172 N18 O66	2826.0729, 1413.5401, 942.6958
**G2 (2COOH)**	C112 H170 N18 O67	2840.0521, 1420.5297, 947.3556
**G2F**	C118 H184 N18 O69	2958.1515, 1479.5794, 986.722
**G2F (CHO)**	C118 H182 N18 O69	2956.1359, 1478.5716, 986.0501
**G2F (2CHO)**	C118 H180 N18 O69	2954.1202, 1477.5637, 985.3783
**G2F (COOH)**	C118 H182 N18 O70	2972.1308, 1486.569, 991.3818
**G2F (2COOH)**	C118 H180 N18 O71	2986.1101, 1493.5587, 996.0415
**G2F (CHO, COOH)**	C118 H180 N18 O70	2970.1151, 1485.5612, 990.7099

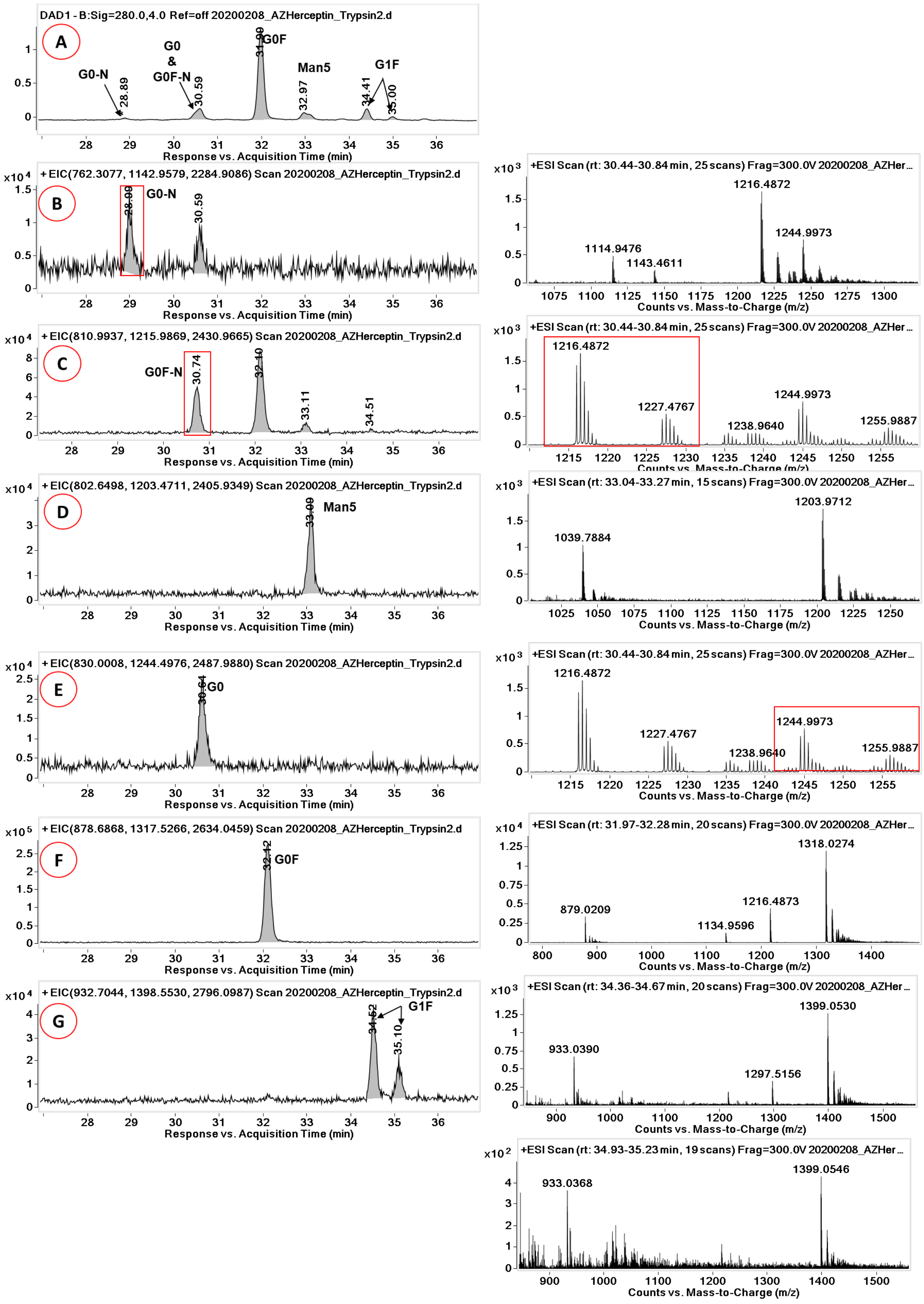

Figure S3. Extracted Ion Chromatograms (EICs) of tryptic digest HILIC/MS data from Trastuzumab **1**. Panels **b-g** show EICs and corresponding MS sections of detected glycoforms (**G0-N**, **G0F-N & G0**, **Man5**, **G0F**, and **G1F**, respectively).

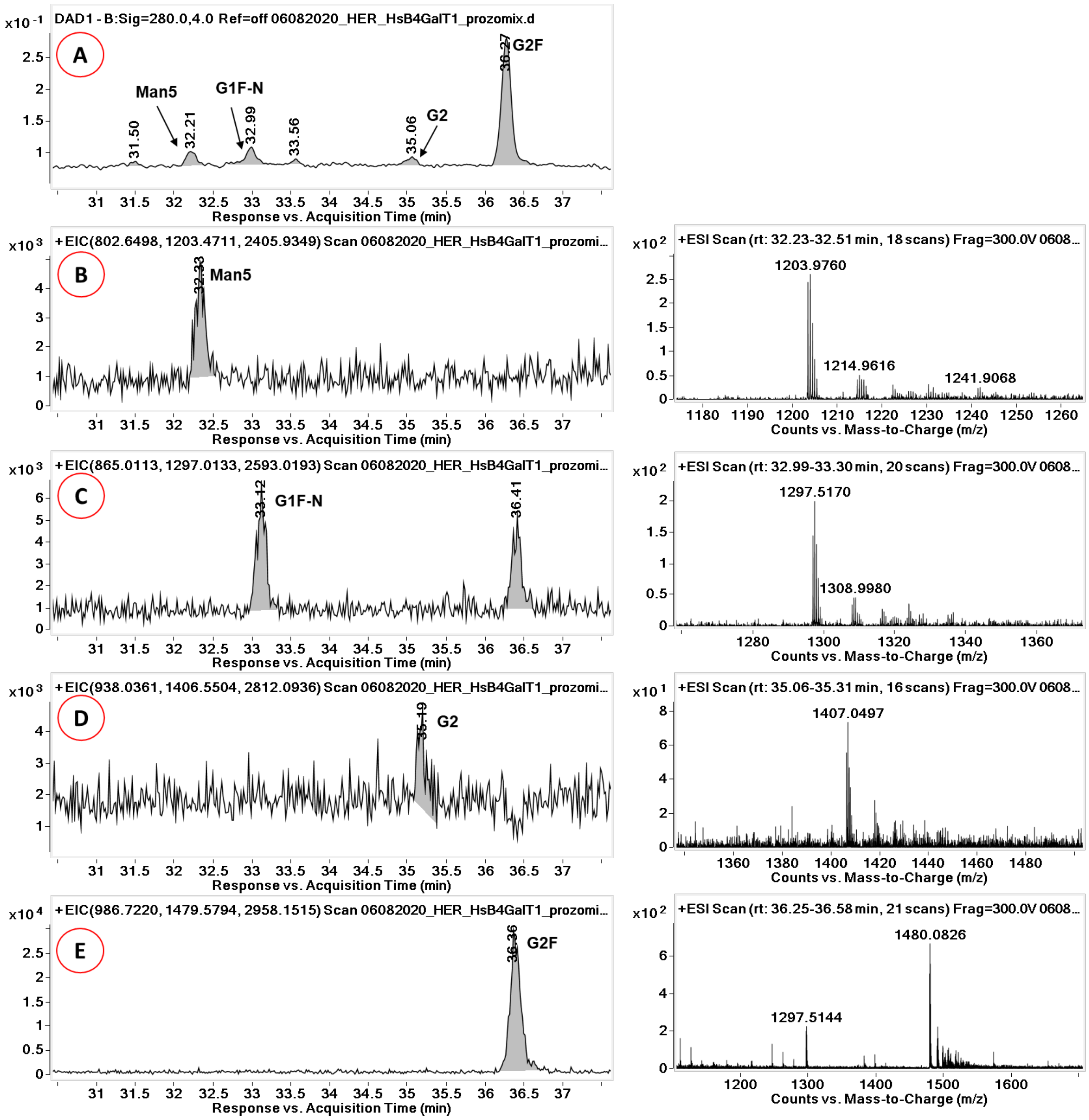

Figure S4. Extracted Ion Chromatograms (EICs) of tryptic digest HILIC/MS data from galactosylated Trastuzumab **2**. Panels **b-e** show EICs and corresponding MS sections of detected glycoforms (**Man**, **G1F-N**, **G2**, and **G2F**, respectively).

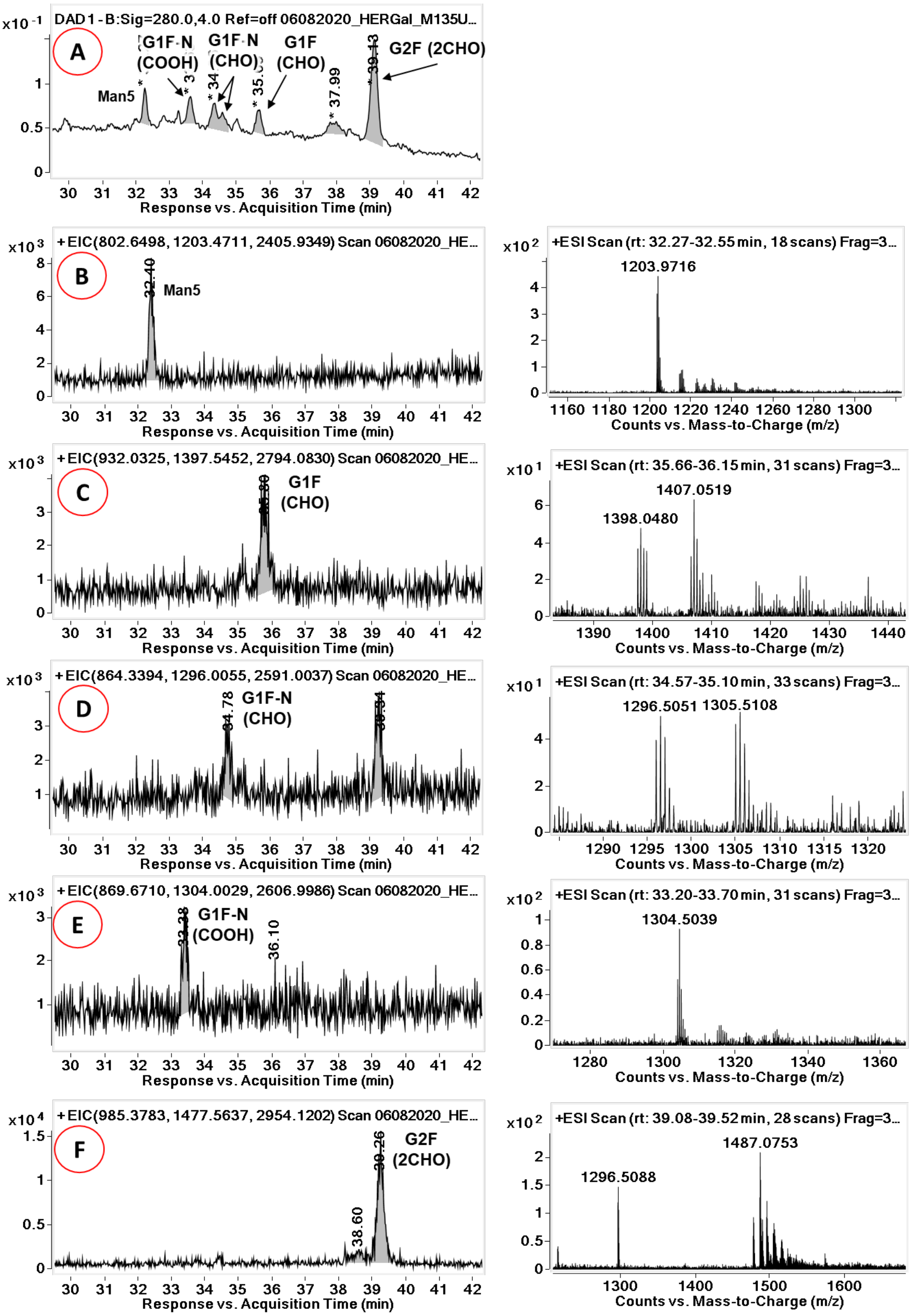

Figure S5. Extracted Ion Chromatograms (EICs) of tryptic digest HILIC/MS data from oxidized Trastuzumab **3**. Panels **b-f** show EICs and corresponding MS sections of detected glycoforms (**Man5**, **G1F (CHO)**, **G1F-N (CHO)**, **G1F-N (COOH)** and **G2F (2CHO)**, respectively).

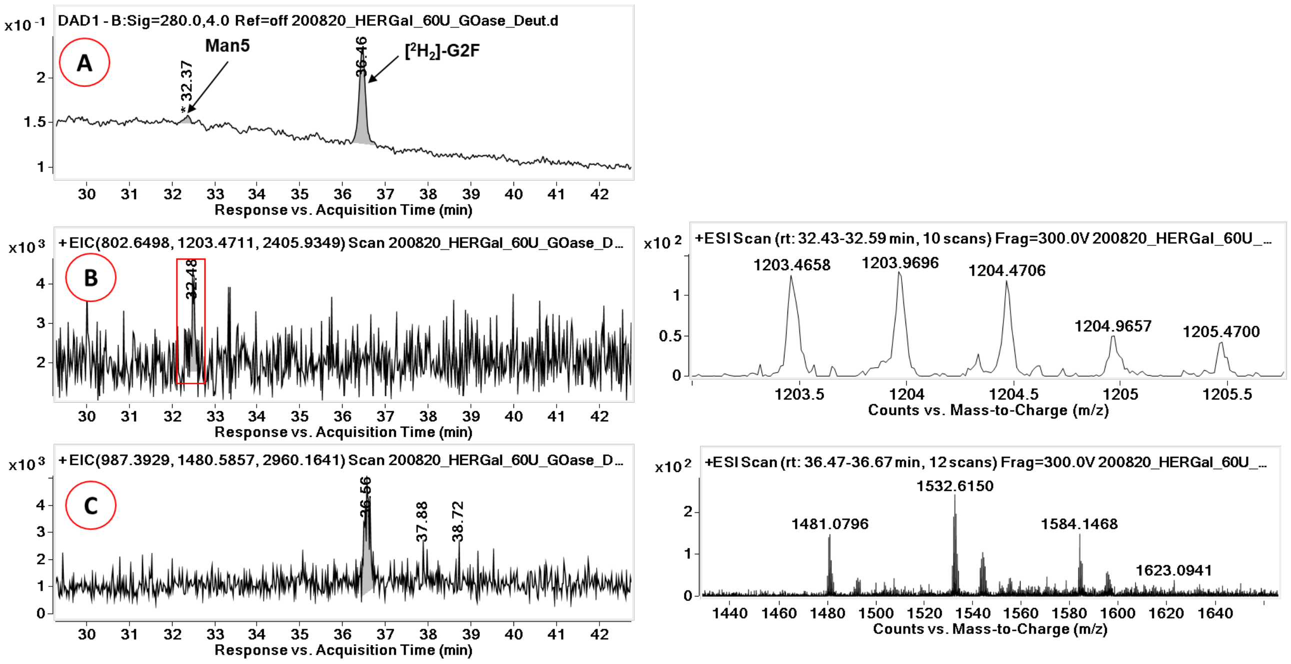

Figure S6. Extracted Ion Chromatograms (EICs) of tryptic digest HILIC/MS data from deuteriated Trastuzumab [^{2}H4]-**3**. Panels **b** and **c** show EIC and corresponding MS section of detected glycoforms (**Man5** and **[^{2}H$_2$]-G2F**, respectively).

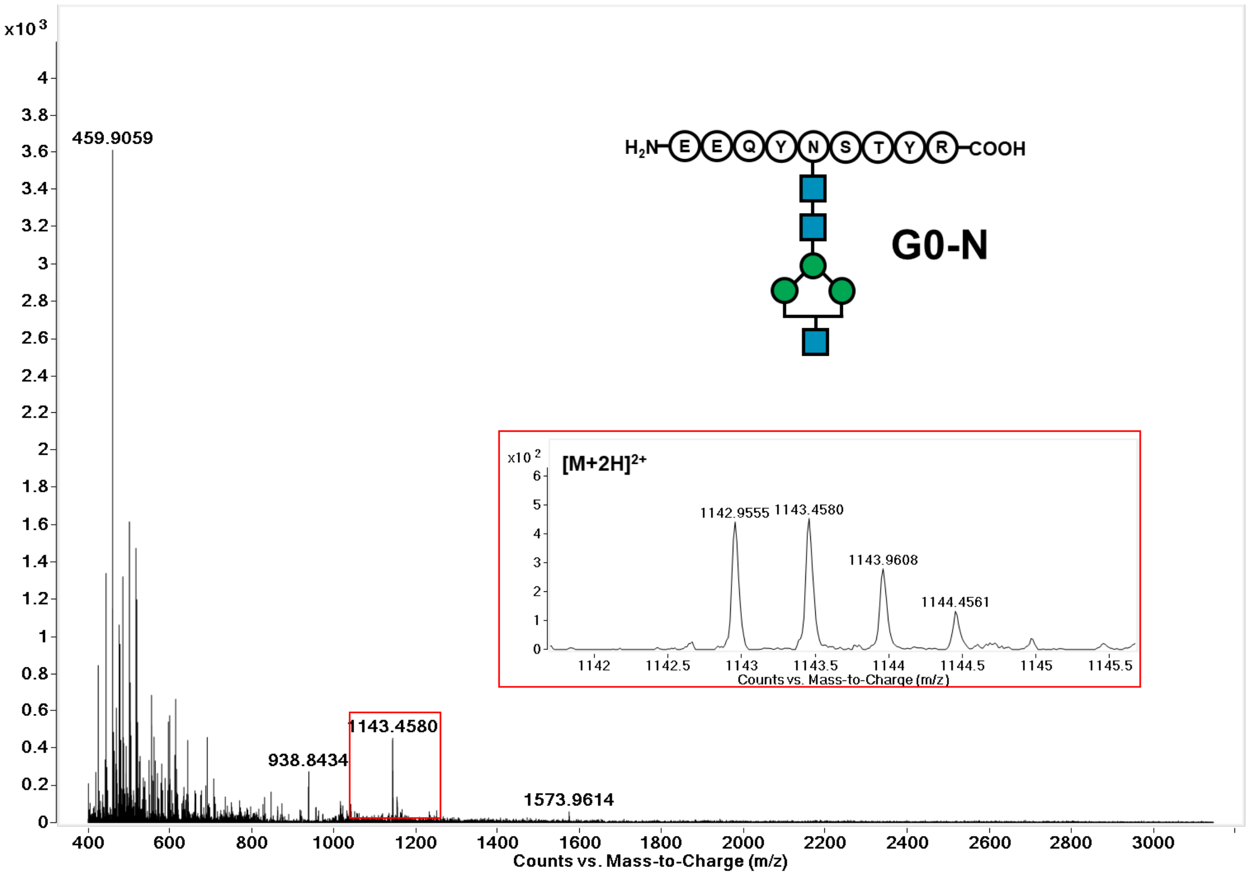

Figure S7. Positive ion MS spectrum of **G0-N** N-glycopeptide fragment from Trastuzumab tryptic digest. Theor. $[M+2H]^{2+}$ 1142.9579, found 1142.9555.

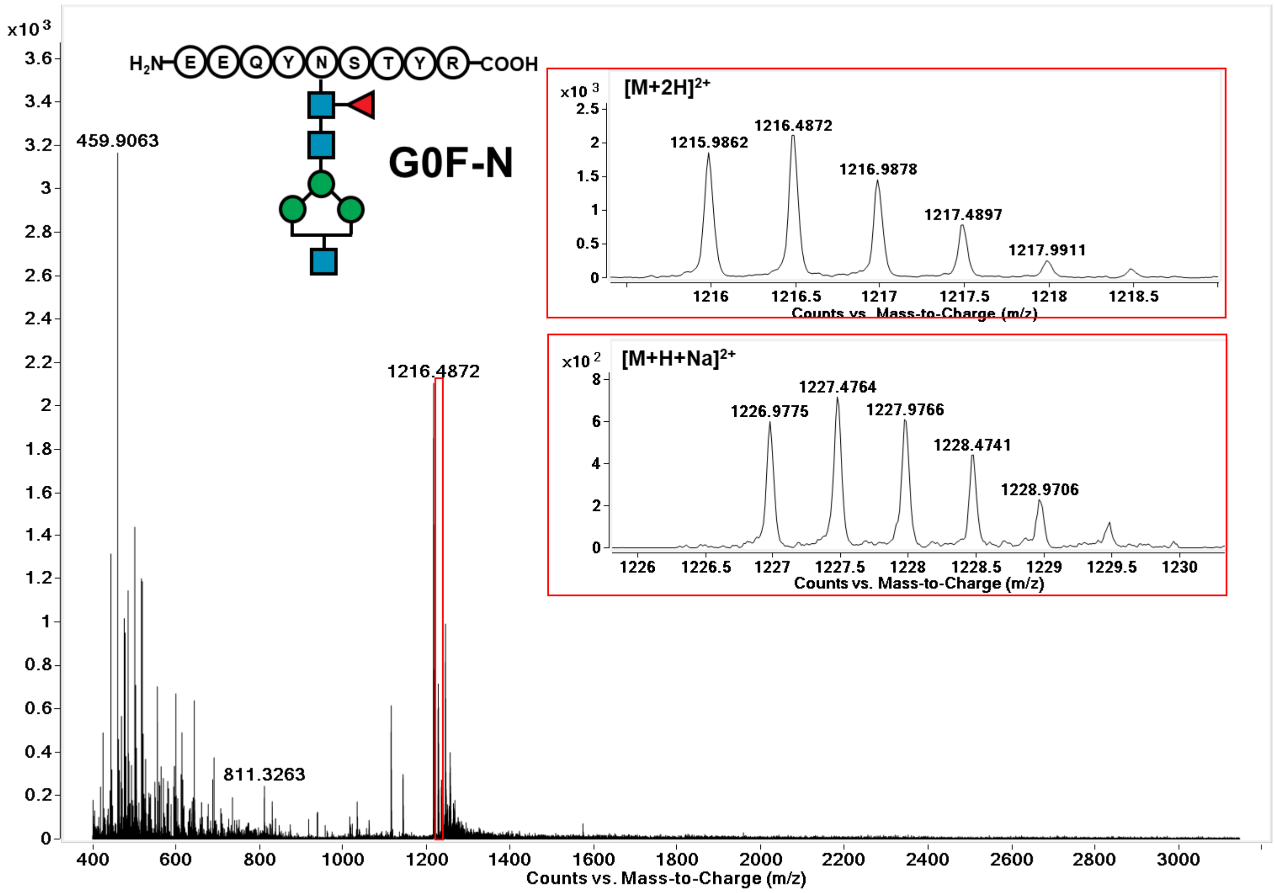

Figure S8. Positive ion MS spectrum of **G0F-N** N-glycopeptide fragment from Trastuzumab tryptic digest. Theor. $[M+2H]^{2+}$ 1215.9869, found 1215.9862; theor. $[M+H+Na]^{2+}$ 1226.9779, found 1226.9775.

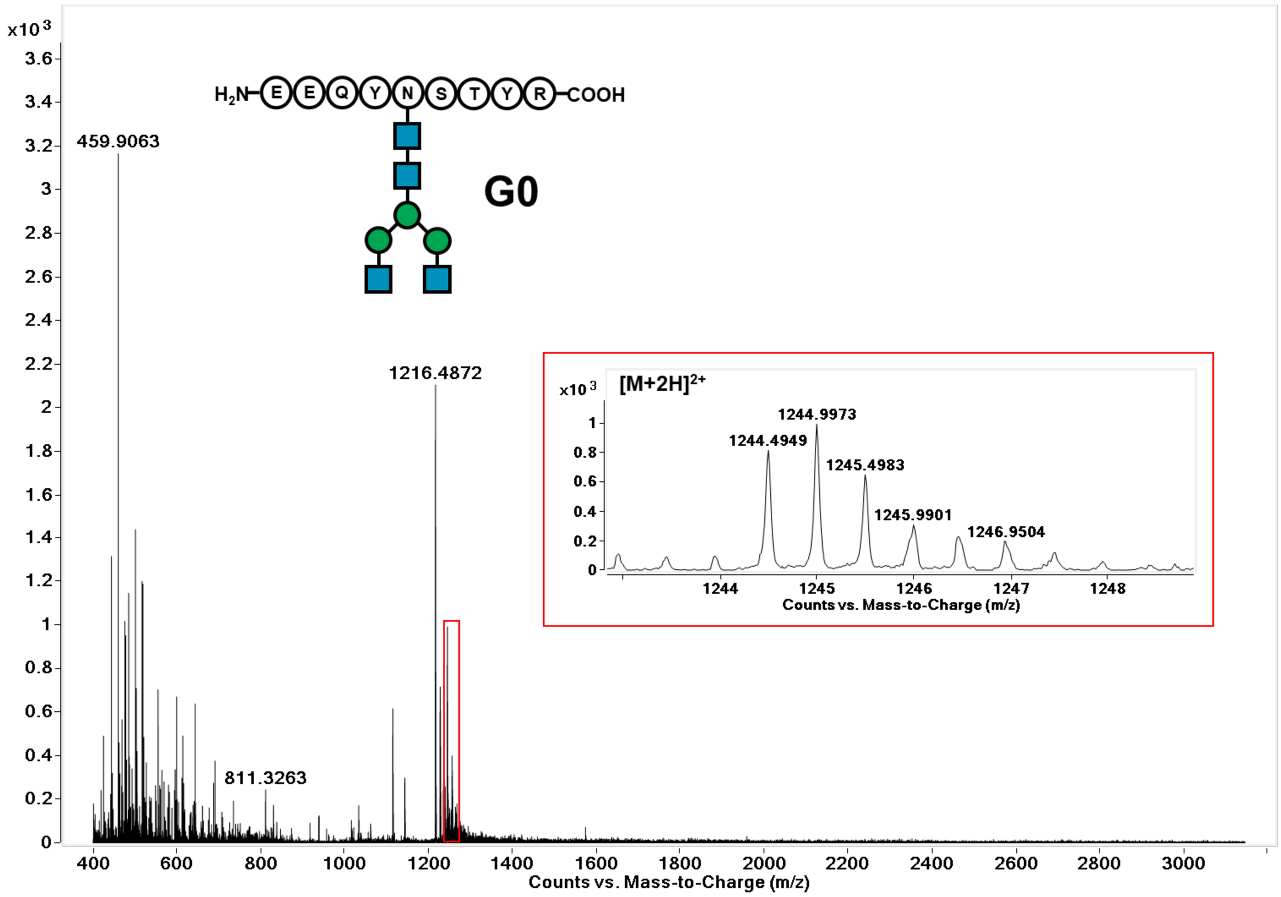

Figure S9. Positive ion MS spectrum of **G0** N-glycopeptide fragment from Trastuzumab tryptic digest. Theor. $[M+2H]^{2+}$ 1244.4976, found 1244.4949.

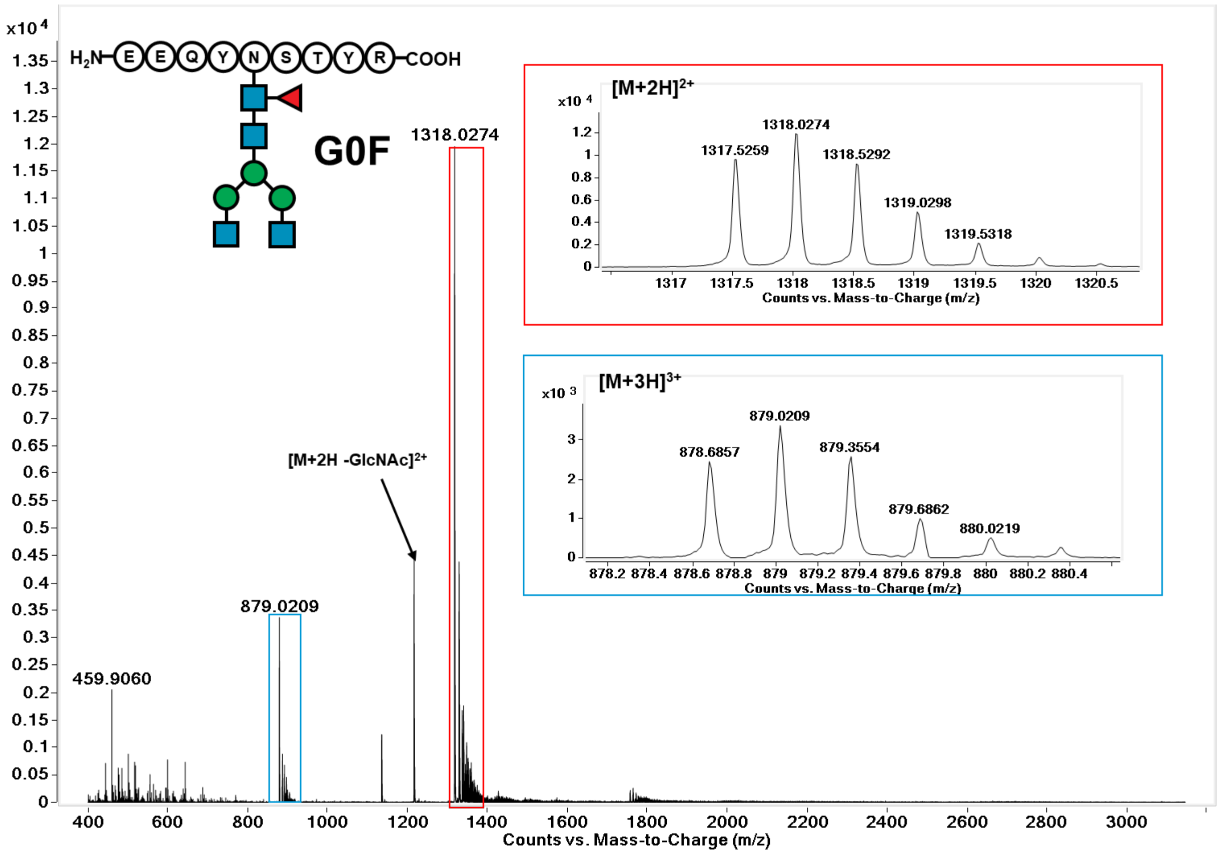

Figure S10. Positive ion MS spectrum of **G0F** N-glycopeptide fragment from Trastuzumab tryptic digest. Theor. $[M+3H]^{2+}$ 878.6868, found 878.6857; theor. $[M+2H]^{2+}$ 1317.5266, found 1317.5229.

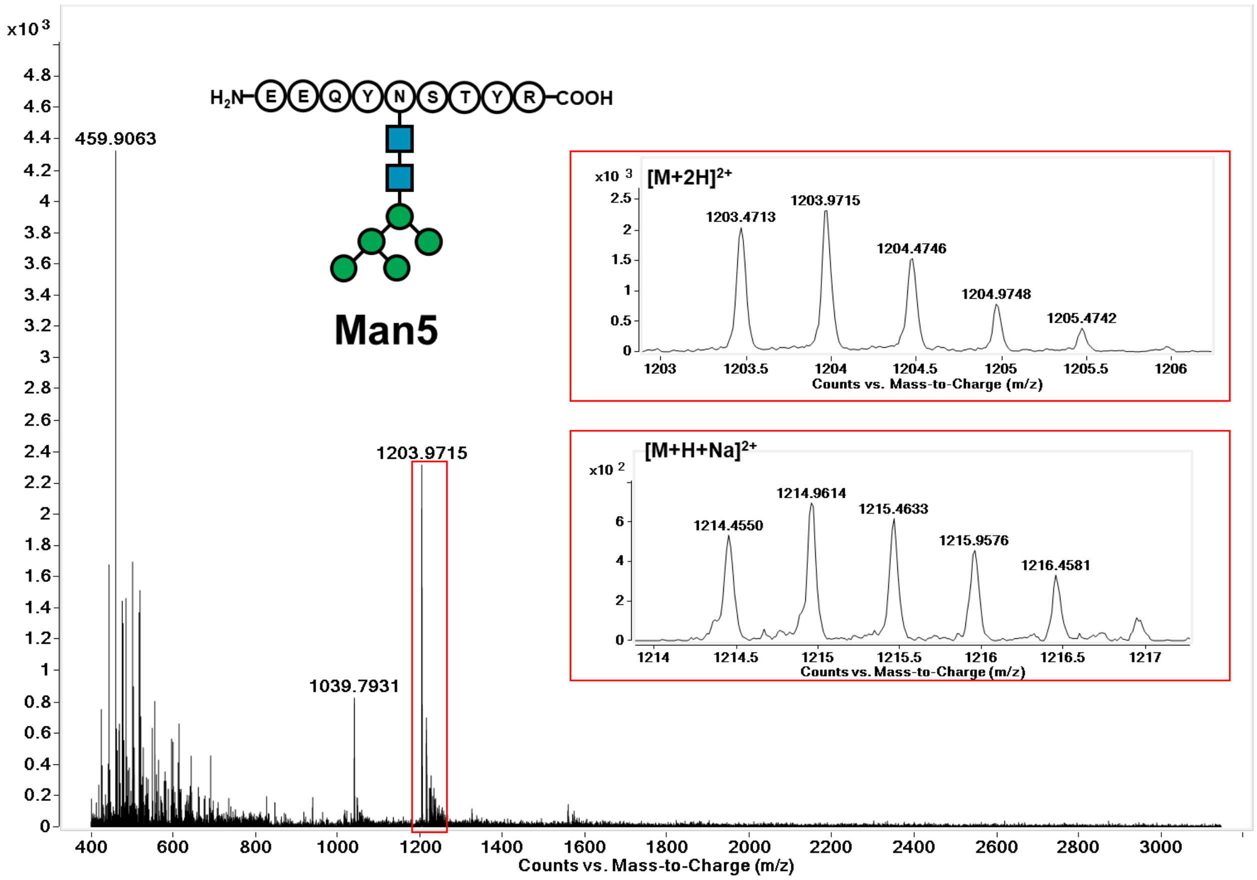

Figure S11. Positive ion MS raw spectrum of **Man5** N-glycopeptide fragment from Trastuzumab tryptic digest. Theor. $[M+2H]^{2+}$ 1203.4711, found 1203.4713; theor. $[M+H+Na]^{2+}$ 1214.4620, found 1214.4550.

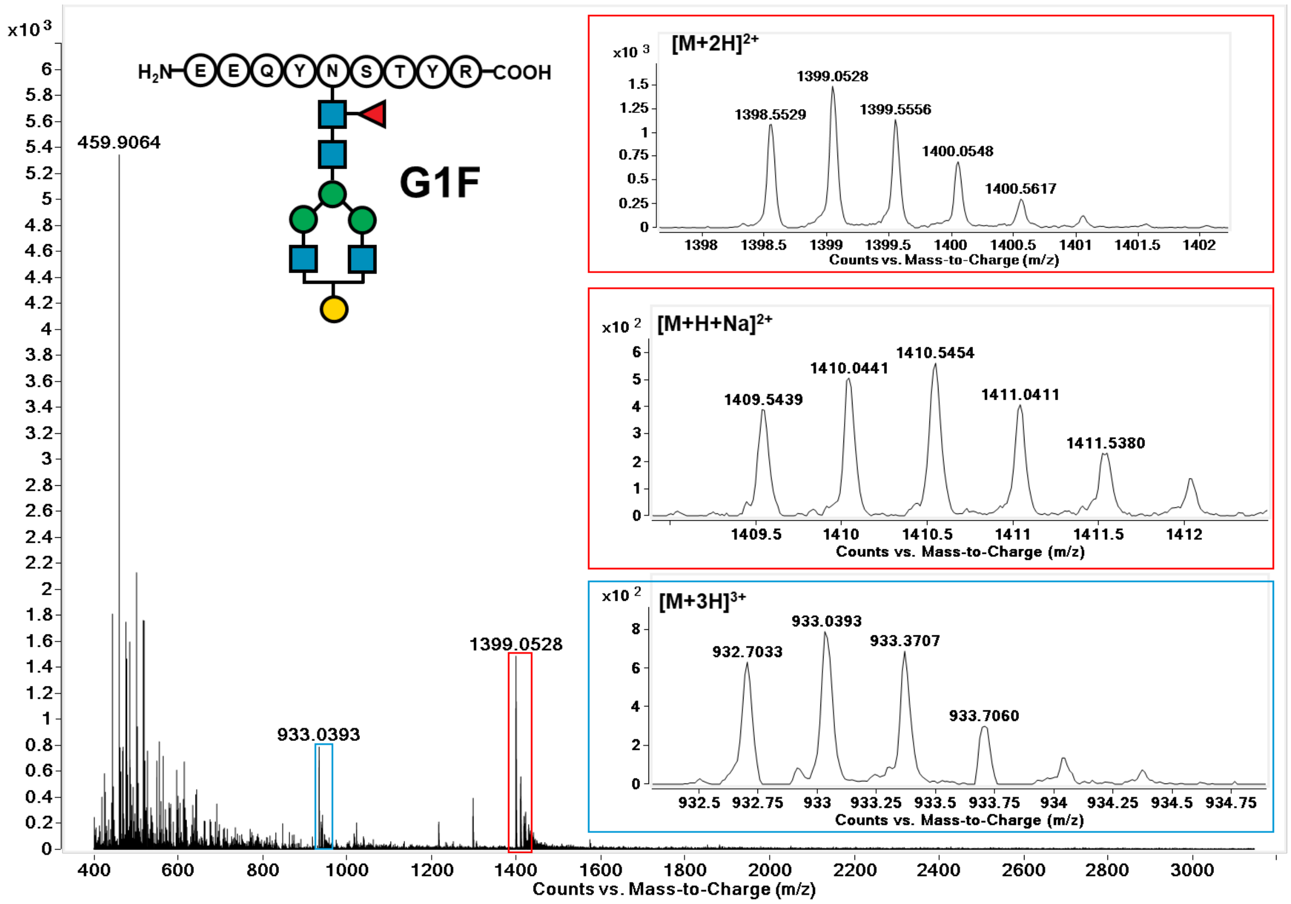

Figure S12. Positive ion MS raw spectrum of **G1F** N-glycopeptide fragment from Trastuzumab tryptic digest. Theor. $[M+2H]^{2+}$ 1398.5530, found 1398.5529; theor. $[M+H+Na]^{2+}$ 1409.5440, found 1409.5439; theor. $[M+3H]^{3+}$ 932.7044, found 932.7033

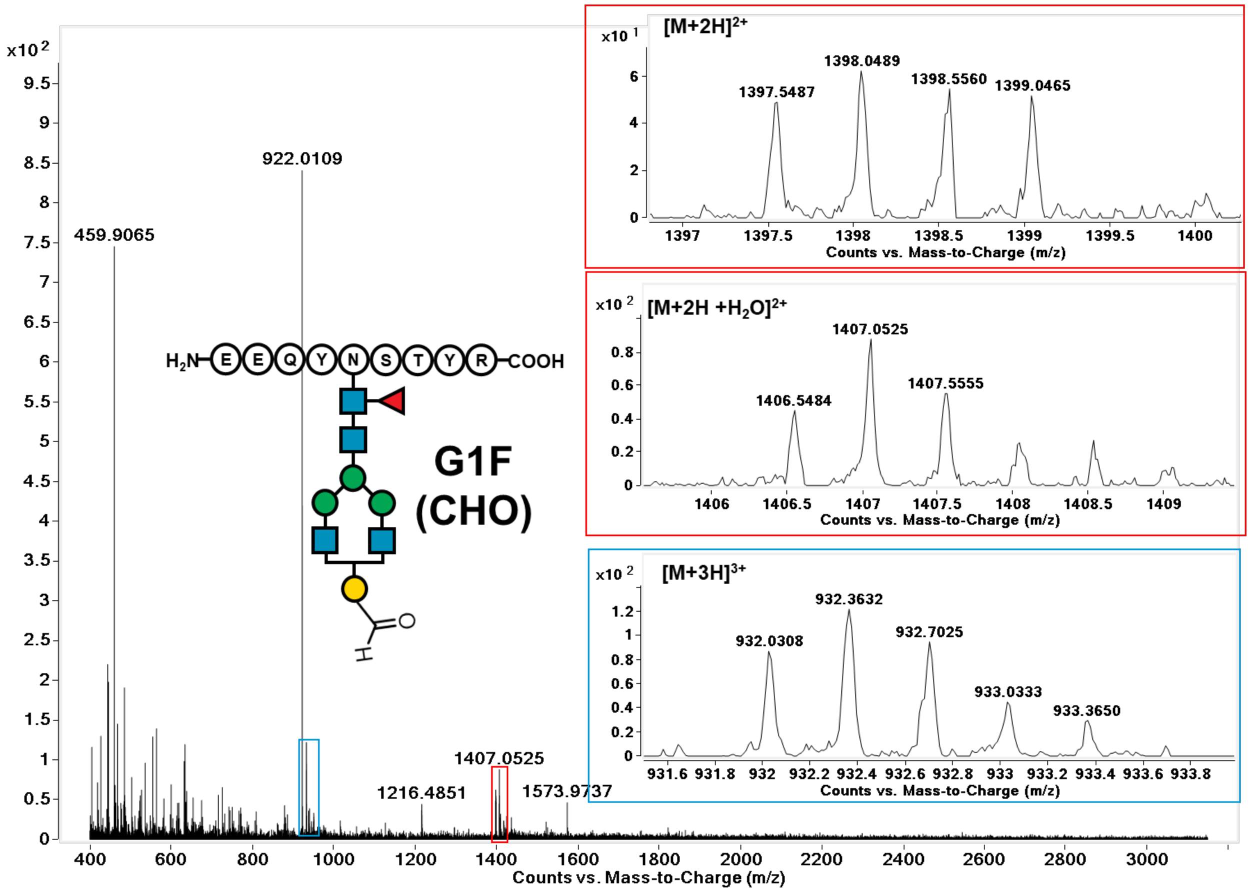

Figure S13. Positive ion MS raw spectrum of **G1F (CHO)** N-glycopeptide fragment from oxidized Trastuzumab tryptic digest. Theor. $[M+2H]^{2+}$ 1397.5452, found 1397.5487; theor. $[M+2H+H_2O]^{2+}$ 1406.5504, found 1406.5484; theor. $[M+3H]^{3+}$ 932.0325, found 932.0308

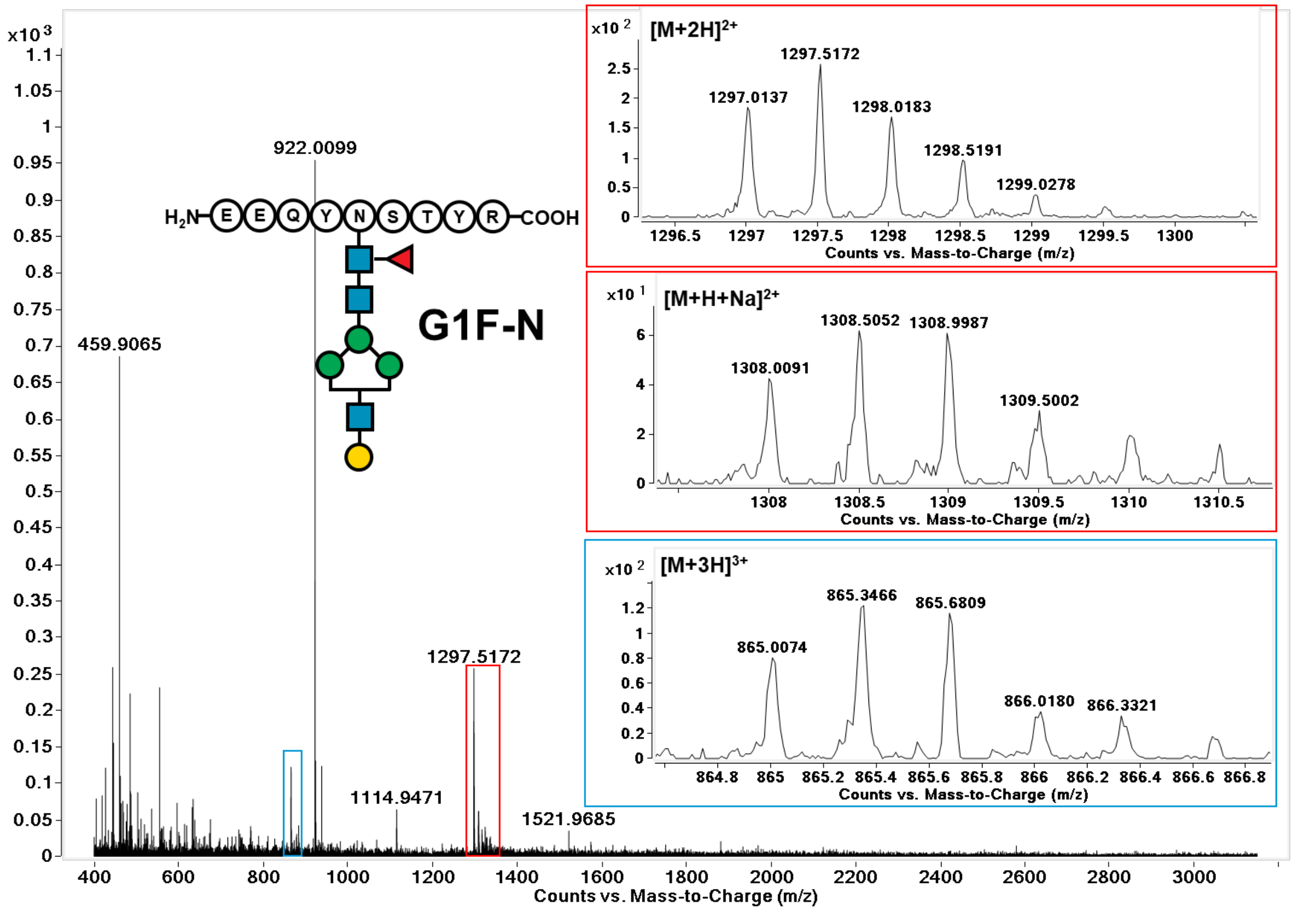

Figure S14. Positive ion MS raw spectrum of **G1F-N** N-glycopeptide fragment from Trastuzumab tryptic digest. Theor. $[M+2H]^{2+}$ 1297.0133, found 1297.0137; theor. $[M+H+Na]^{2+}$ 1308.0043, found 1308.0091; theor. $[M+3H]^{3+}$ 865.0113, found 865.0074.

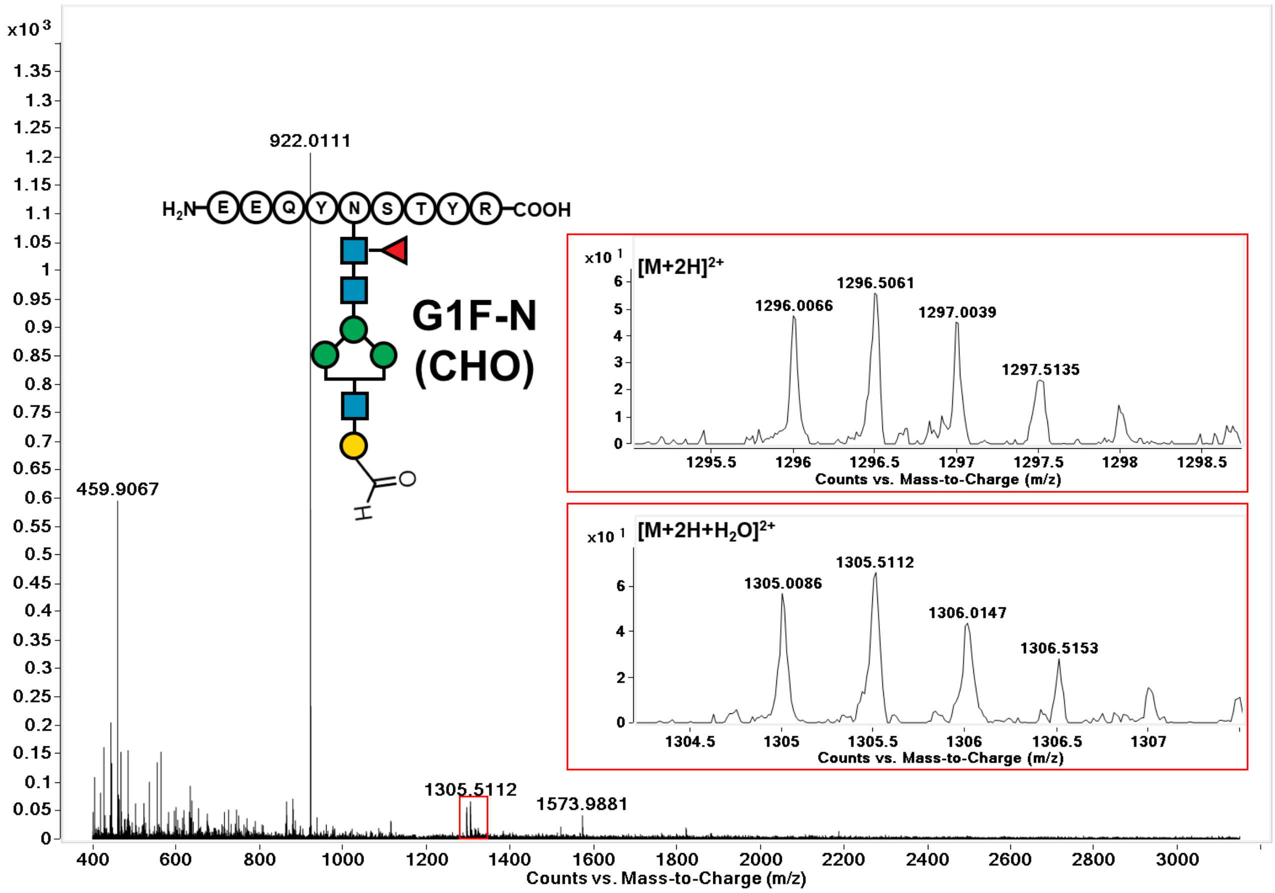

Figure S15. Positive ion MS raw spectrum of **G1F-N (CHO)** N-glycopeptide fragment from Trastuzumab tryptic digest. Theor. $[M+2H]^{2+}$ 1296.0055, found 1296.0066; theor. $[M+2H +H_2O]^{2+}$ 1305.0108, found 1305.0086.

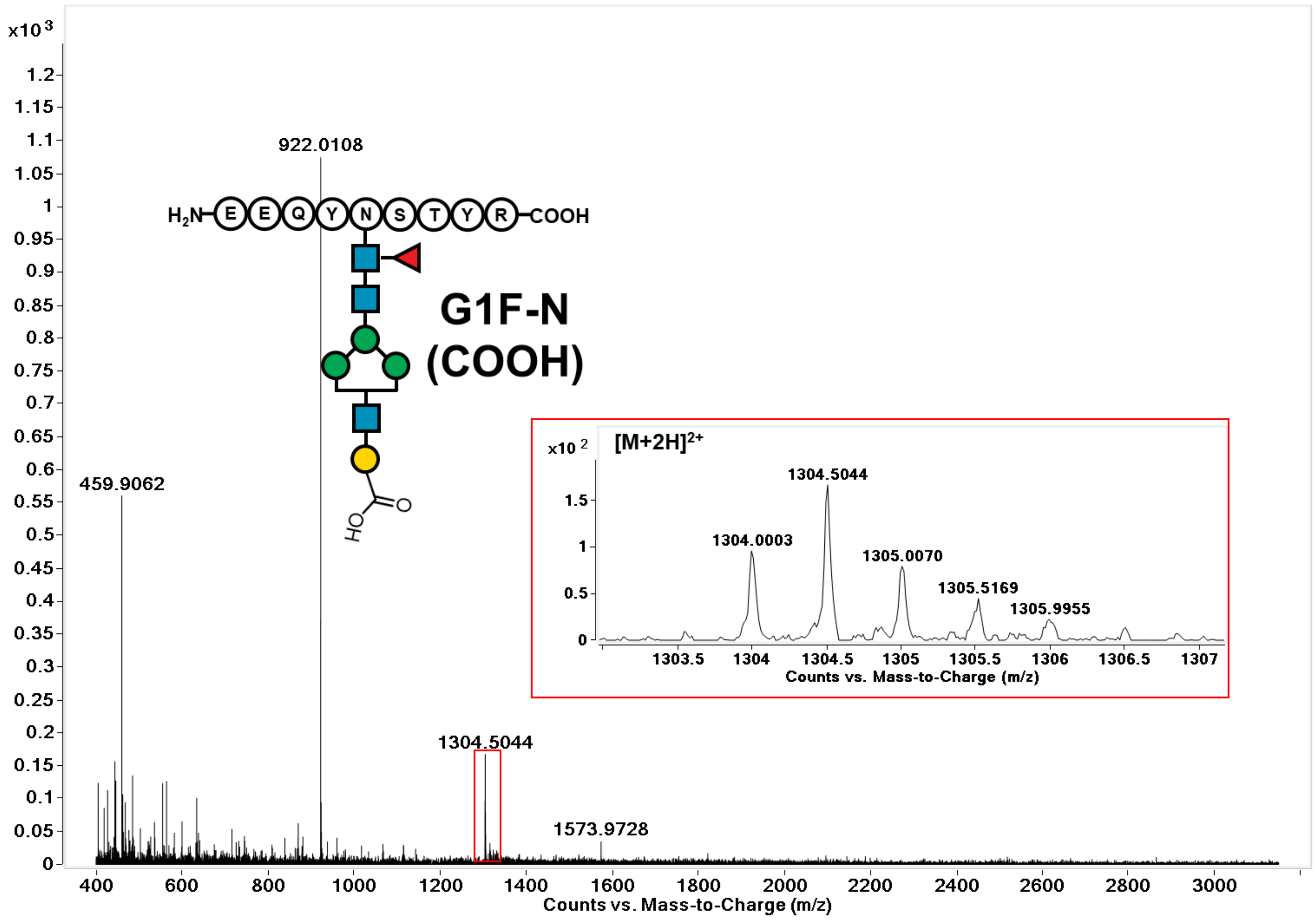

Figure S16. Positive ion MS raw spectrum of **G1F-N (COOH)** N-glycopeptide fragment from Trastuzumab tryptic digest. Theor. $[M+2H]^{2+}$ 1304.0029, found 1304.0003.

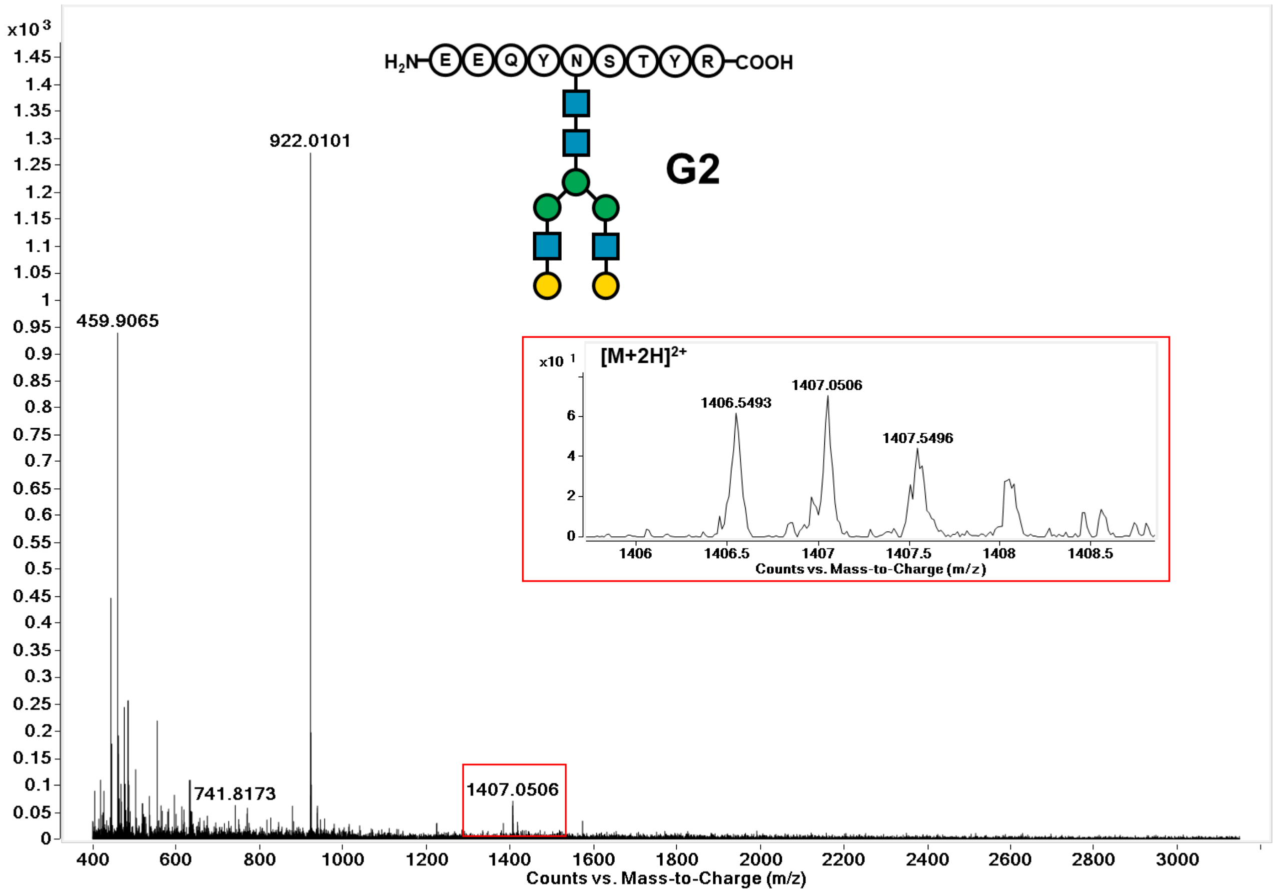

Figure S17. Positive ion MS raw spectrum of **G2** N-glycopeptide fragment from Trastuzumab tryptic digest. Theor. $[M+2H]^{2+}$ 1406.5504, found 1406.5493.

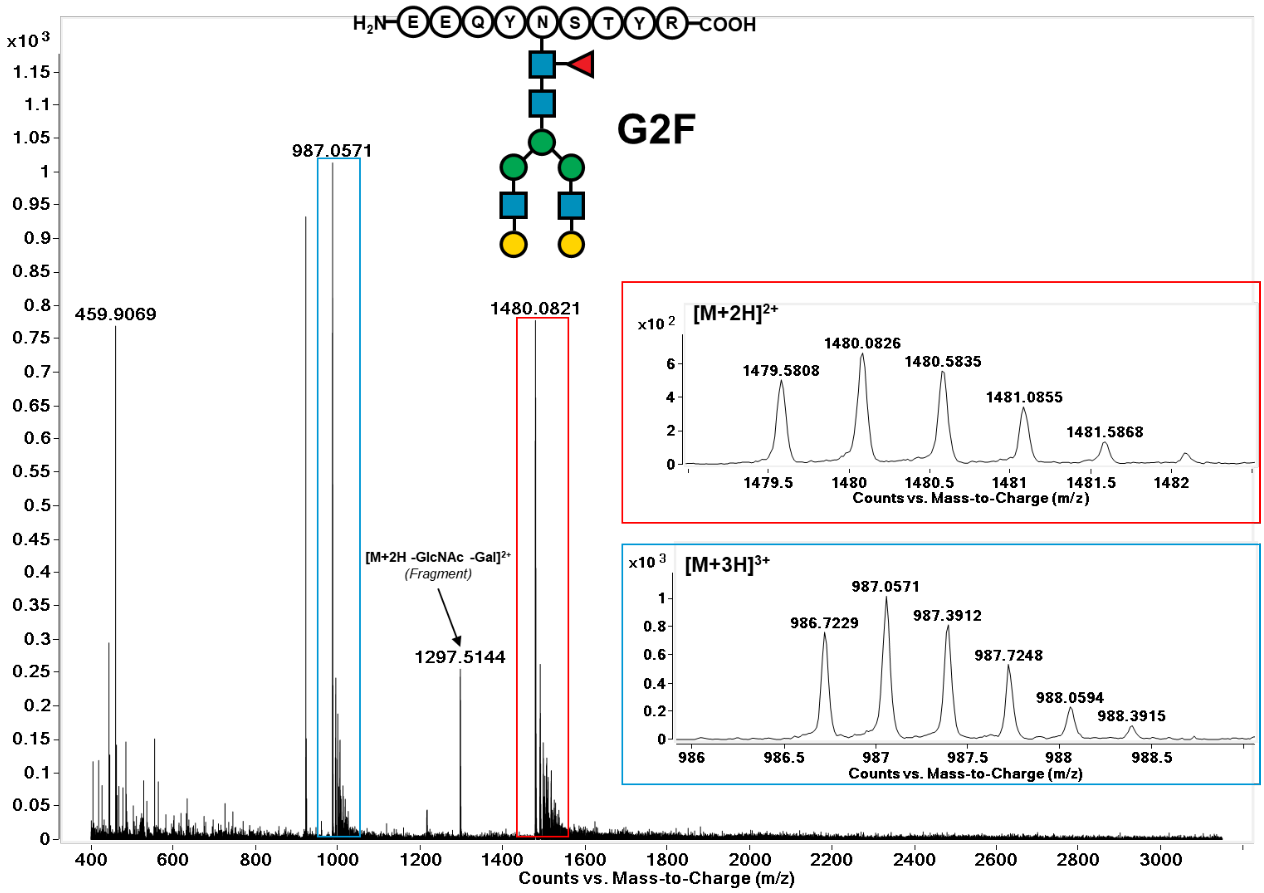

Figure S18. Positive ion MS raw spectrum of **G2F** N-glycopeptide fragment from Trastuzumab tryptic digest. Theor. $[M+2H]^{2+}$ 1479.5794, found 1479.5808; $[M+3H]^{3+}$ 986.7220, found 986.7229.

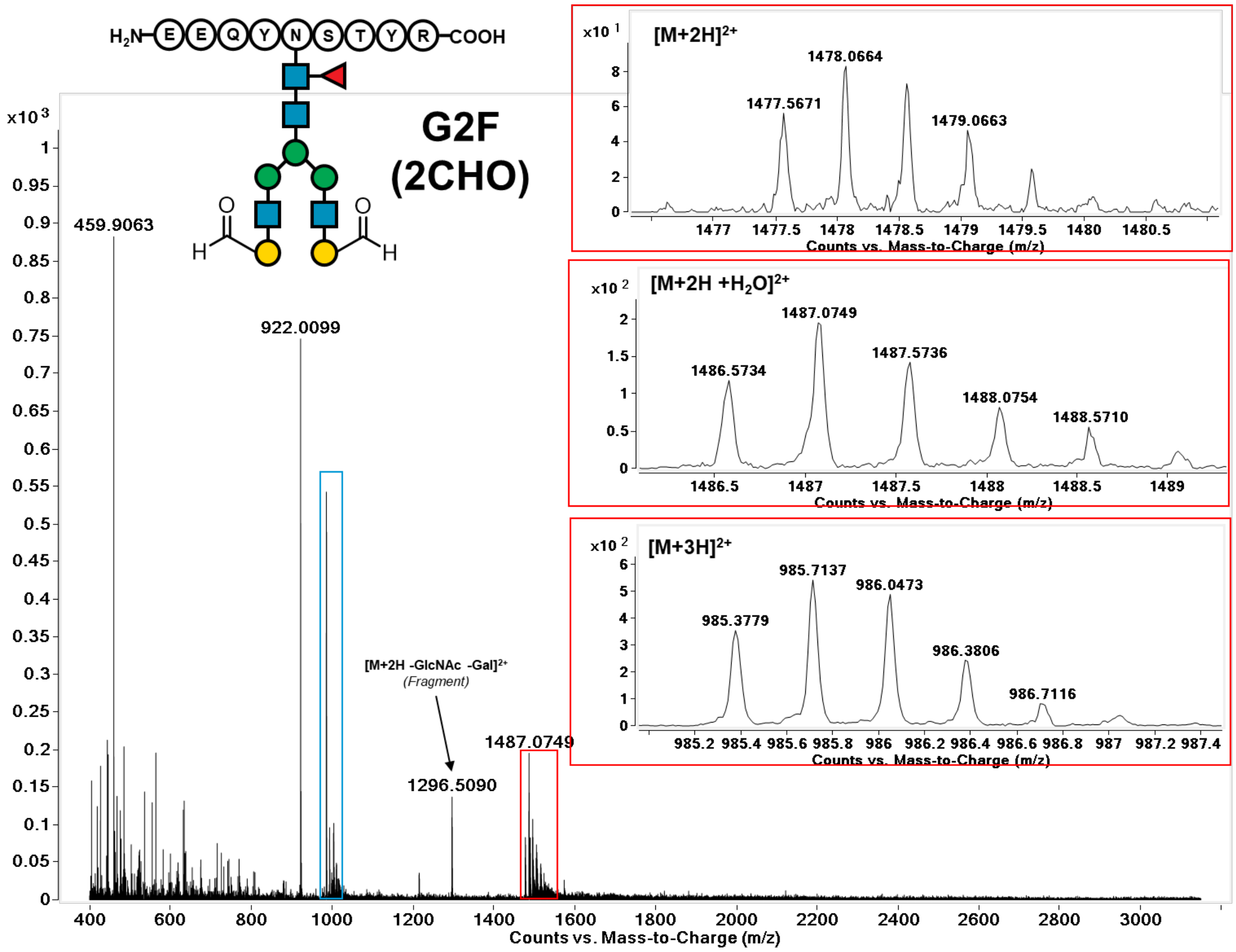

Figure S19. Positive ion MS raw spectrum of **G2F (2CHO)** N-glycopeptide fragment from Trastuzumab tryptic digest. Theor. $[M+2H]^{2+}$ 1477.5637, found 1477.5671; $[M+2H +H_2O]^{2+}$ 1486.5690, found 1486.5734; $[M+3H]^{3+}$ 985.3783, found 985.3779.

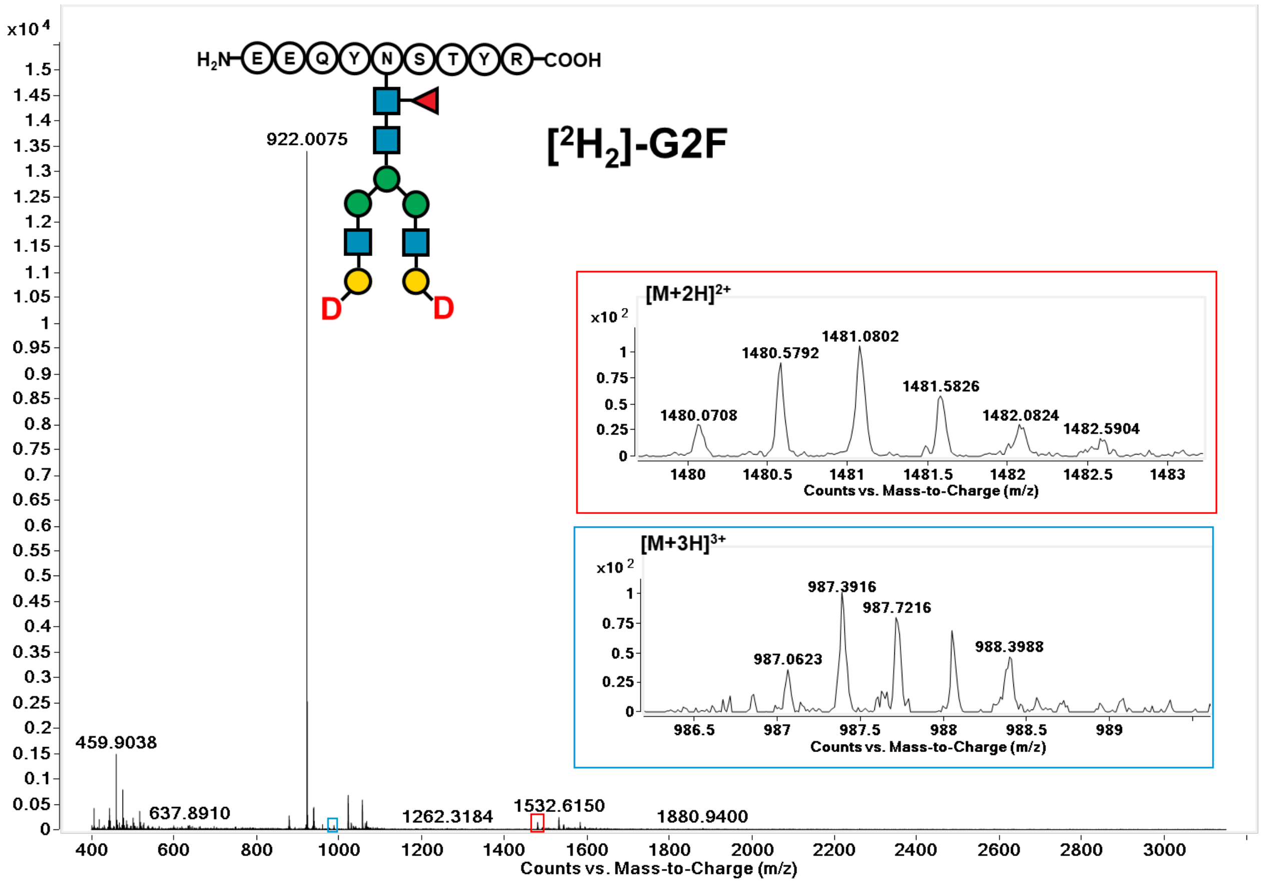

Figure S20. Positive ion MS raw spectrum of **[$^2H_2$]-G2F** N-glycopeptide fragment from Trastuzumab tryptic digest. Theor. $[M+2H]^{2+}$ 1480.5857, found 1480.5792; theor. $[M+3H]^{3+}$ 987.3929, found 987.3916.

Antibody Fc/2 fragment analysis *via* RPLC/MS

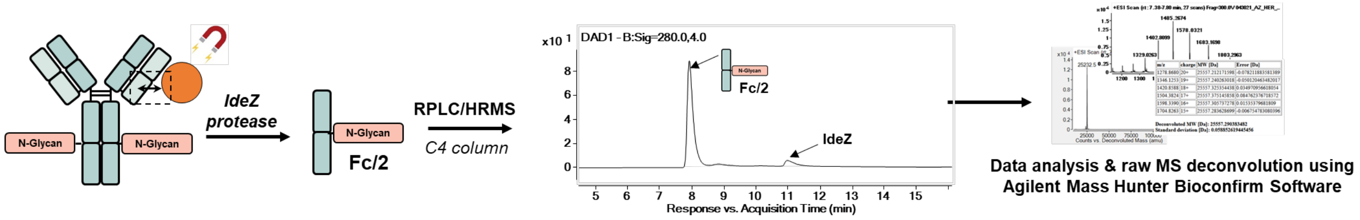

Figure S21. Schematic representation of the general protocol for RPLC/MS analysis of Trastuzumab Fc/2 fragment.

Preparation of Fc/2 fragment. 25-50 µg of antibody sample was immobilized on Protein L magnetic beads. Magnetic beads were washed with 50 mM phosphate pH = 7.5 three times using a magnetic stand. Beads were then treated with 0.5 µL of IdeZ protease (NEB) in ~15 µL 50 mM phosphate pH = 7.5 for 1 h at 37 °C. After incubation, buffer containing the Fc/2 fragment released by the protease was separated from magnetic beads and transferred into a MS vial for RPLC/MS analysis. Deglycosylation of Fc/2 fragment was performed by adding 1 µL of Rapid PNGase F (NEB) directly to the MS vial followed by incubation at 37 °C for 1 h prior to RPLC/MS analysis.

Reverse-phase liquid chromatography / mass spectrometry (RPLC/MS) of the antibody Fc/2 fragment. Data was acquired using an Agilent 6520 Ion Mobility LC/Q-TOF system in positive mode (reference mass 922.00979800) equipped with a LC Agilent 1290 Infinity system and Waters ACQUITY UPLC glycoprotein BEH C4 (300 Å, 1.7 µm) column. Column flow and temperature were set at 0.2 mL/min and 45 °C, respectively. Fc/2 fragment (prepared as described above) was separated via a linear gradient of 20% to 95% acetonitrile in 30 min.

Raw RPLC/MS data was processed using MassHunter Workstation Software Version B.08.00 (Agilent Technologies, Inc. 2016). Raw MS spectrum associated to Fc/2 fragment was deconvoluted using BioConfirm (Version 10.0, Build 10.010136.0) using Maximum Entropy deconvolution algorithm (m/z range 1000.000-2300.000). The resulting deconvoluted spectrum was inspected and assigned manually (Table S3 & Figures S21-27).

Table S3. Molecular formulas and corresponding theoretical masses of Fc/2 fragments generated from mAb partial digestion with IdeZ protease.

Fc/2 Glycoform	Molecular formula	Average theoretical mass (neutral)
**Non-glycosylated**	C1066 H1644 N282 O322 S7	23786.6
**Deglycosylated (PNGase F)**	C1066 H1643 N281 O323 S7	23787.6
**G0-N**	C1066 H1644 N282 O322 S7	24882.6
**Man5**	C1112 H1720 N284 O357 S7	25003.7
**G0F-N**	C1114 H1723 N285 O356 S7	25028.7
**G0**	C1116 H1726 N286 O357 S7	25085.8
**G1F-N**	C1120 H1732 N284 O362 S7	25191.9
**G1F-N (CHO)**	C1120 H1730 N284 O362 S7	25189.9
**G0F**	C1122 H1736 N286 O361 S7	25231.9
**G1**	C1122 H1736 N286 O362 S7	25247.9
**G1 (CHO)**	C1122 H1734 N286 O362 S7	25245.9
**G2**	C1128 H1746 N286 O367 S7	25410.1
**G2 (1CHO)**	C1128 H1744 N286 O367 S7	25408.1
**G2 (2CHO)**	C1128 H1742 N286 O367 S7	25406.0
**G2F**	C1134 H1756 N286 O371 S7	25556.2
**G2F (1CHO)**	C1134 H1754 N286 O371 S7	25554.2
**G2F (2CHO)**	C1134 H1752 N286 O371 S7	25552.2
**G1F-N (2 N_3)**	C1120 H1730 N284 O362 S7	25189.9
**G1 (2 N_3)**	C1150 H1780 N298 O371 S7	25940.7
**G2 (2 N_3)**	C1156 H1790 N298 O376 S7	26102.8
**G2 (4 N_3)**	C1184 H1834 N310 O385 S7	26795.5
**G1F (2 N_3)**	C1156 H1790 N298 O375 S7	26086.8
**G2F (2 N_3)**	C1162 H1800 N298 O380 S7	26248.9
**G2F (4 N_3)**	C1190 H1844 N310 O389 S7	26941.7
**G1F-N (2 N_3 + 2 DBCO-TAMRA)**	C1228 H1844 N294 O382 S7	27062.0

**G1 (2 N_3 + 2 DBCO-TAMRA)**	C1258 H1894 N308 O391 S7	27812.8
**G2 (2 N_3 + 2 DBCO-TAMRA)**	C1264 H1904 N308 O396 S7	27974.9
**G2 (4 N_3 + 4 DBCO-TAMRA)**	C1400 H2062 N330 O425 S7	30539.8
**G1F (2 N_3 + 2 DBCO-TAMRA)**	C1264 H1904 N308 O395 S7	27958.9
**G2F (2 N_3 + 2 DBCO-TAMRA)**	C1270 H1914 N308 O400 S7	28121.1
**G2F (4 N_3 + 4 DBCO-TAMRA)**	C1406 H2072 N330 O429 S7	30685.9

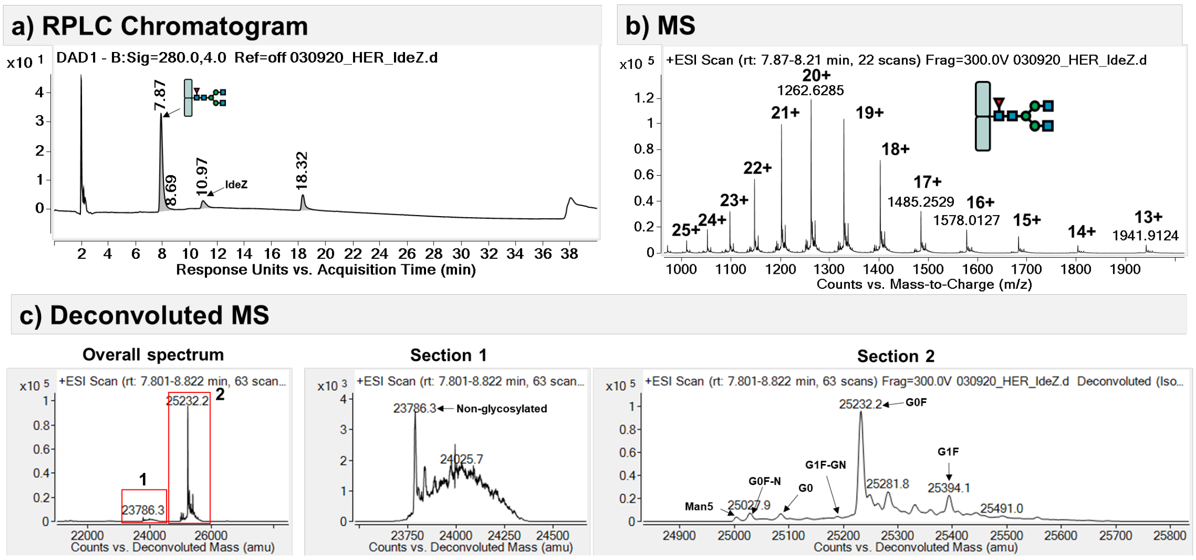

Figure S22. Analysis of Fc/2 fragment generated from native Trastuzumab **1**.

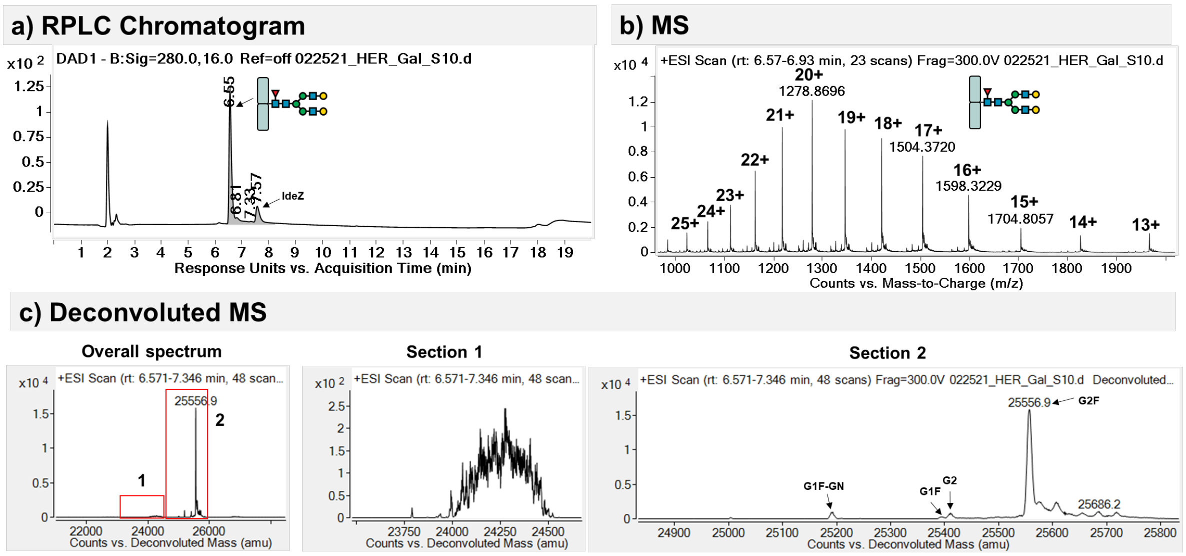

Figure S23. Analysis of Fc/2 fragment generated from galactosylated Trastuzumab **2**.

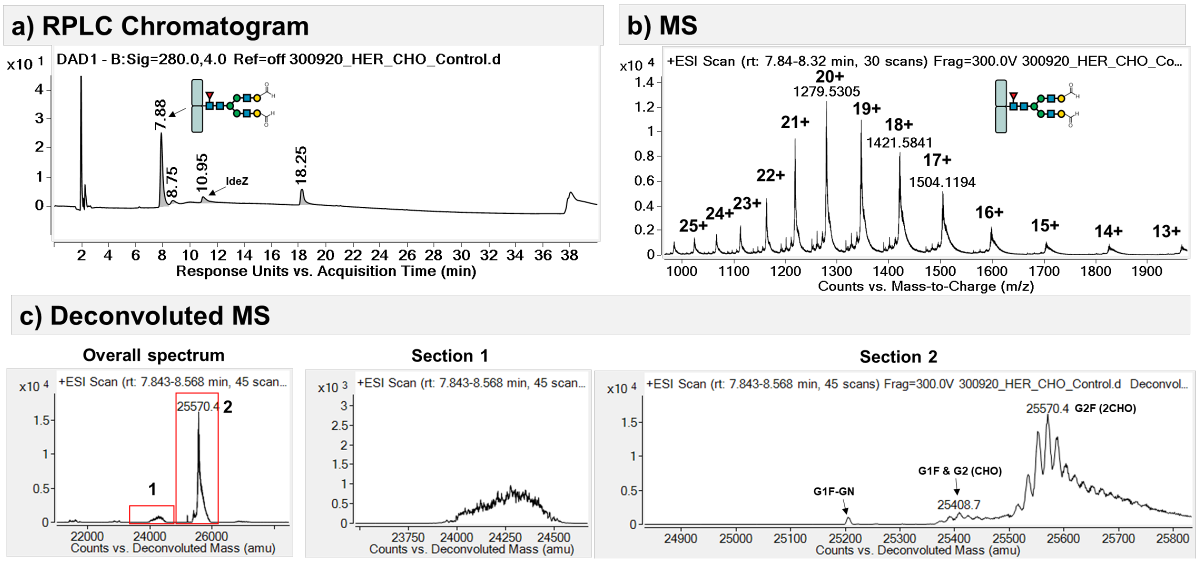

Figure S24. Analysis of Fc/2 fragment generated from oxidized Trastuzumab **3**.

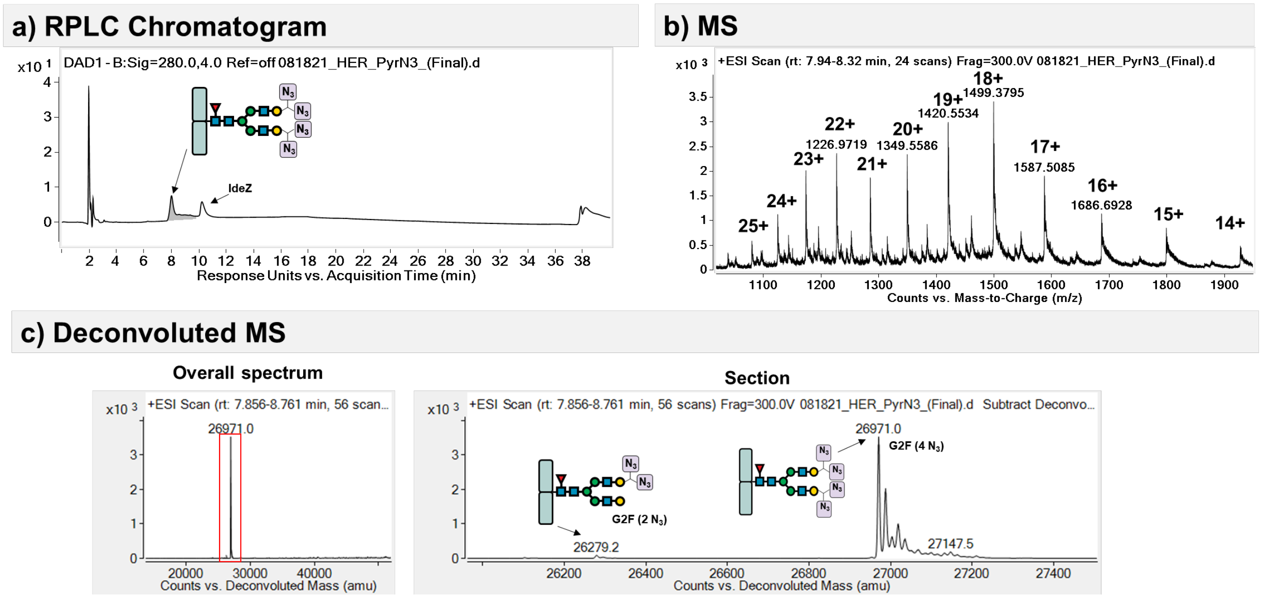

Figure S25. Analysis of Fc/2 fragment generated from azido-Trastuzumab **5**.

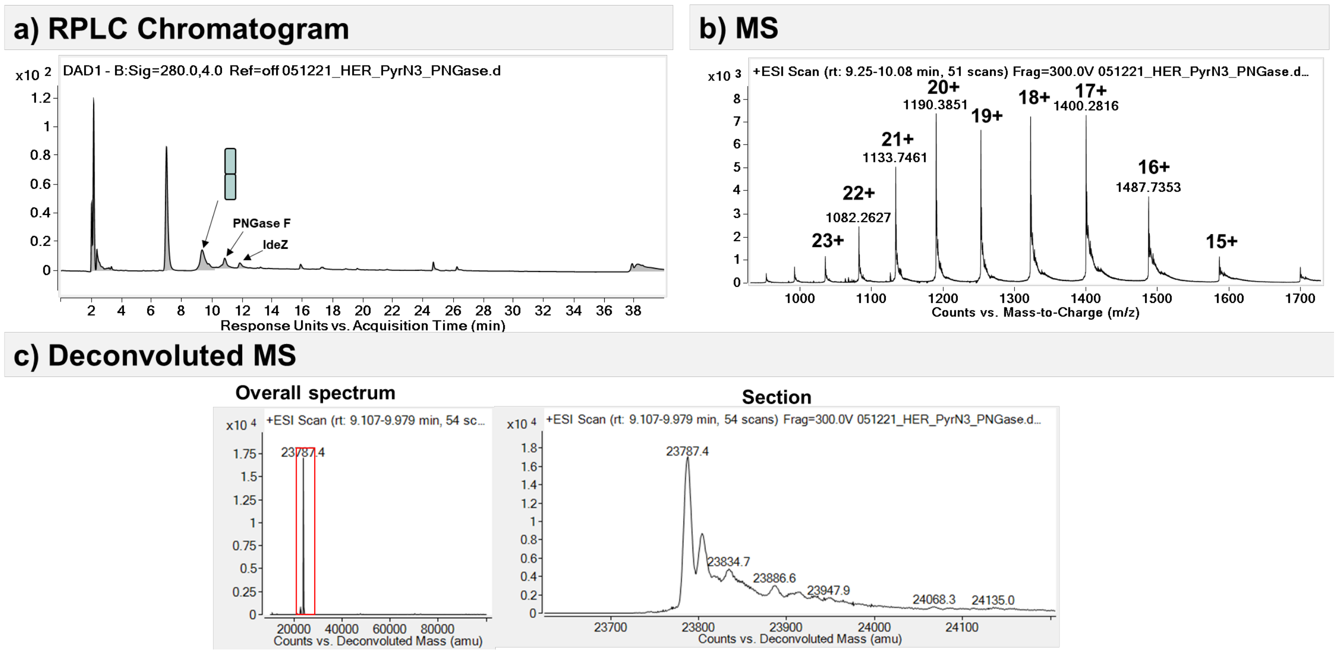

Figure S26. Analysis of Fc/2 fragment generated from azido-Trastuzumab **5** treated with PNGase F.

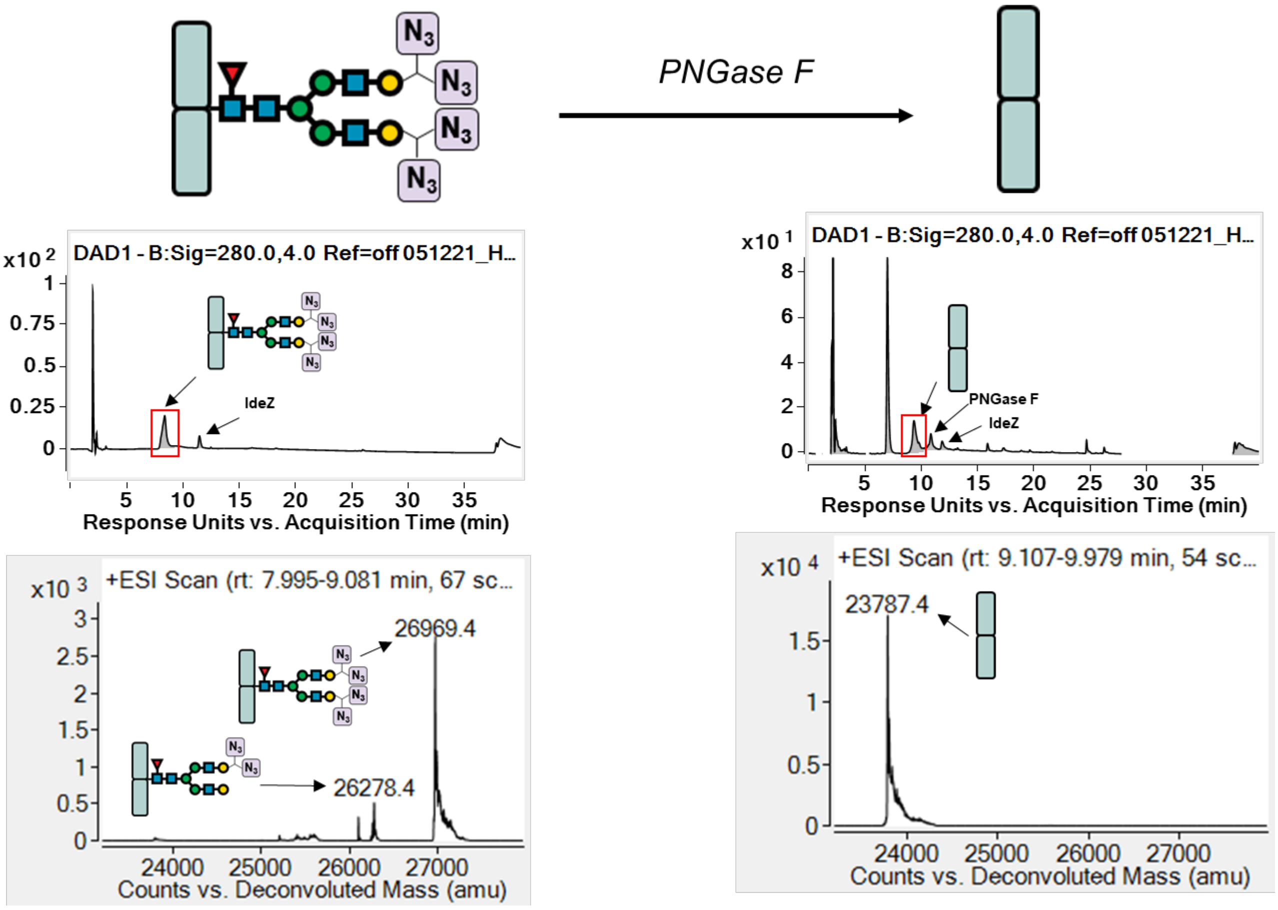

Figure S27. Comparison between azido-Trastuzumab **5** Fc/2 fragment analyses before and after treatment with of PNGase F.

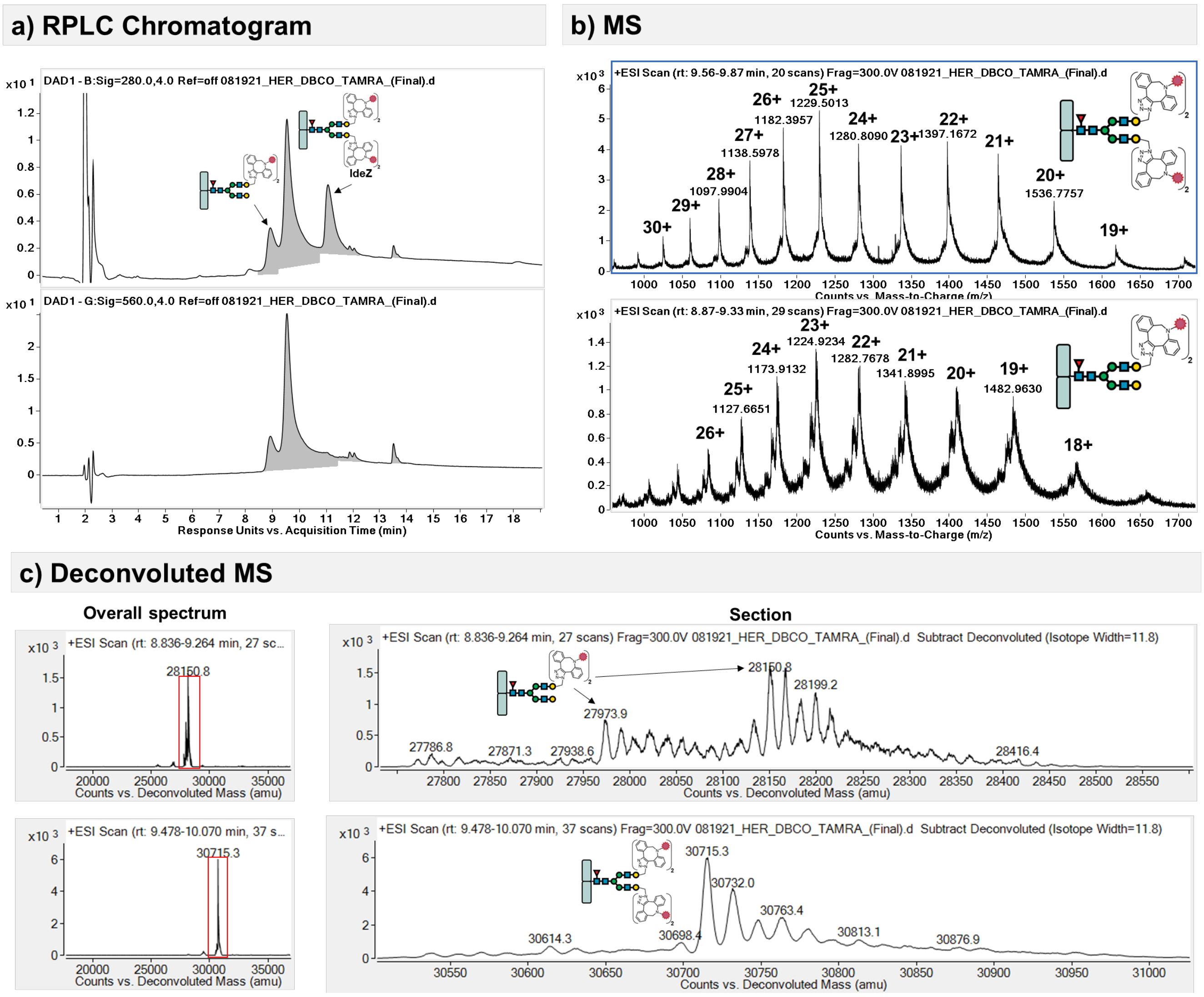

Figure S28. Analysis of Fc/2 fragment generated from Trastuzumab conjugated to DBCO-TAMRA **6**.

Average DAR Estimation. Relative quantification of Fc/2 N-glycan species conjugated to the azido-linker was performed by calculating the ratio of the corresponding integrated areas from the deconvoluted MS spectra of azido-Trastuzumab **5** (Figure S28a). Peaks corresponding to partially (+2 N_3) and fully (+4 N_3) conjugated N-glycan species were confirmed experimentally by comparing deconvoluted MS data between azido-Trastuzumab **5** and its DBCO-TAMRA conjugated **6** counterpart (Figure S28). All other minor species between ≈ 23876 and ≈ 27200 amu were included into calculation on the assumption of not being conjugated (Table S4).

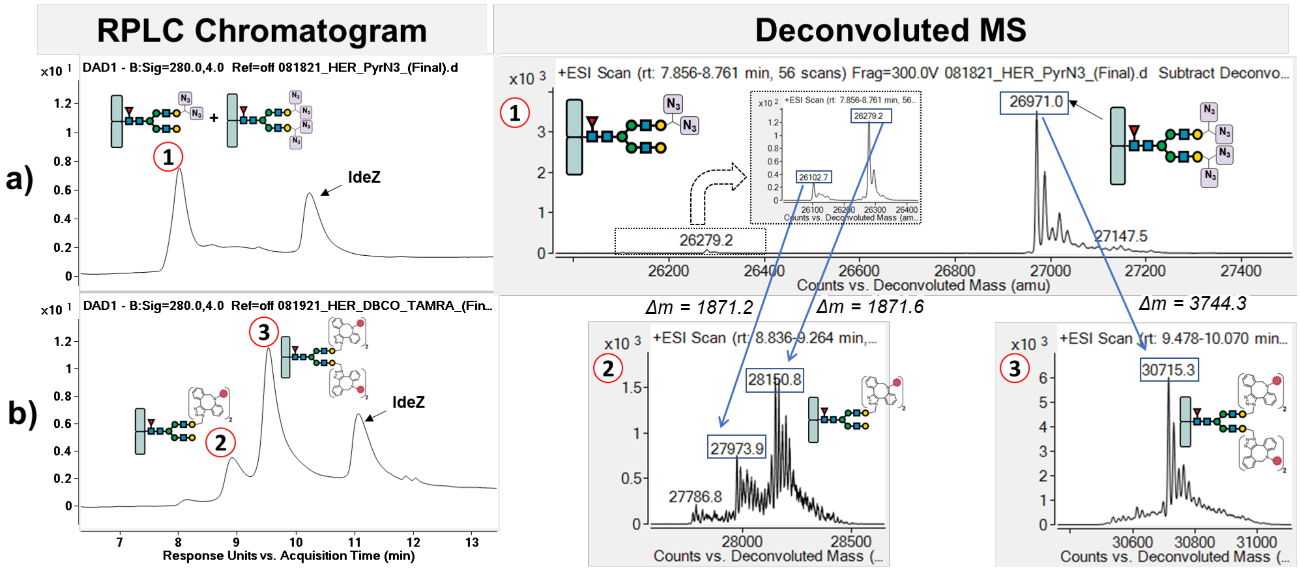

Figure S29. Comparison between Fc/2 fragment analyses generated from **a**) azido-Trastuzumab **5** and **b**) azido-Trastuzumab conjugated to DBCO-TAMRA **6**. While a theoretical mass shift of +936.06 amu is expected for each DBCO-TAMRA conjugated to azido-Trastuzumab, mass increases of ≈ 1872 and 3744 amu are observed in species conjugated to 2 and 4 payloads, respectively.

Table S4. Calculated % abundance of azido-Trastuzumab **5** Fc/2 species derived from deconvoluted MS data.

	**% Abundance**
**Non-conjugated species**	8
**Partially conjugated (+ 2 N_3)**	2
**Fully conjugated (+ 4 N_3)**	90

Estimation of the average DAR for the functionalized intact antibody was therefore calculated using the following formula:

$$Avg\ DAR = \left(\frac{\sum \%\ abundace\ x\ \#N_3}{100}\right) x\ 2 = \left(\frac{(7.6\ x\ 0) + (2.3\ x\ 2) + (90.1\ x\ 4)}{100}\right) x\ 2 = 7.3$$

Synthesis of N-(2-(2-(2-(2-azidoethoxy)ethoxy)ethoxy)ethyl)-2-(3-methyl-5-oxo-4,5-dihydro-1H-pyrazol-1-yl)acetamide (4).

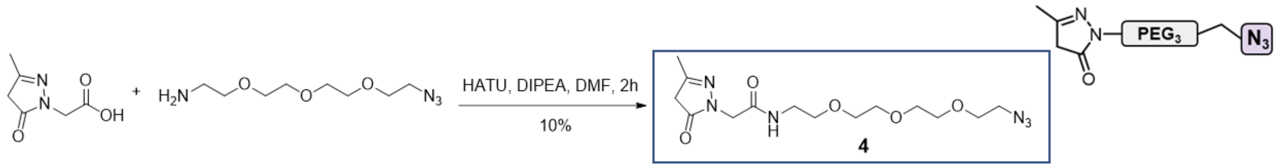

(3-methyl-5-oxo-4,5-dihydro-1H-pyrazol-1-yl)acetic acid hydrochloride (200 mg, 1.04 mmol) was dissolved in 10 mL DMF and mixed with 724 µL of DIPEA (4 eq.). HATU (414 mg, 1.1 mmol, 1.05 eq.) dissolved in 4 mL DMF was added to mixture, followed by addition 11-Azido-3,6,9-trioxaundecan-1-amine (238 mg, 1.1 mmol, 1 eq.) solution in DMF dropwise. The reaction mixture was stirred for 2h, followed by DMF removal under reduced pressure. Crude product was purified by RP-HPLC. Fractions corresponding to the product were confirmed by LC/MS and merged. The final product (39 mg, 0.11 mmol, 10% yield) was obtained as a light brown solid as TFA salt.

^{1}H NMR (400 MHz, DMSO-*d6*) δ 7.84 (t, *J* = 5.6 Hz, 1H), 5.13 (s, 1H), 4.35 (s, 2H), 3.63 – 3.59 (m, 3H), 3.54 (ddt, *J* = 11.3, 4.2, 2.1 Hz, 10H), 3.43 (t, *J* = 5.7 Hz, 2H), 3.39 (dd, *J* = 9.9, 5.1 Hz, 3H), 3.23 (q, *J* = 5.7 Hz, 2H), 2.02 (s, 3H). ^{13}C NMR (101 MHz, DMSO) δ 173.26, 167.67, 167.37, 156.84, 146.94, 87.03, 70.28, 70.24, 70.16, 70.07, 69.73, 69.39, 50.47, 48.37, 46.64, 41.33, 39.18, 39.10, 19.32, 16.93, 14.28.

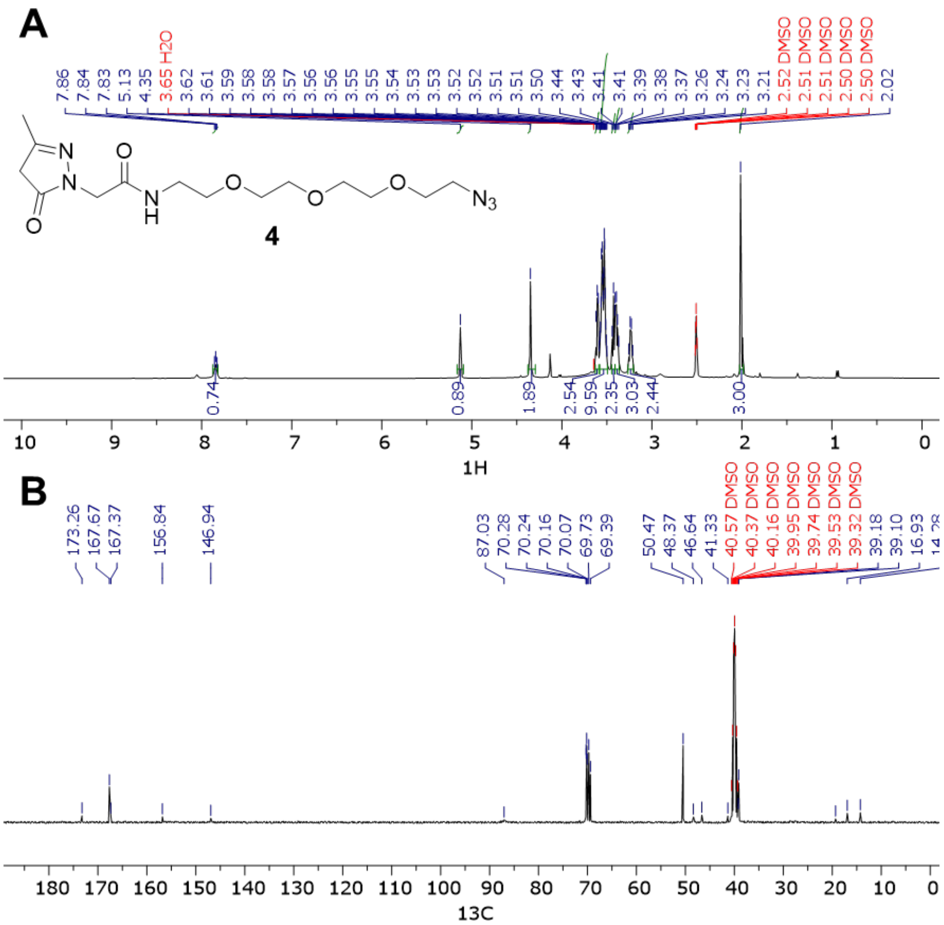
A
7.86 7.84 7.83 5.13 4.35 3.65 H2O 3.62 3.61 3.59 3.58 3.58 3.57 3.56 3.56 3.55 3.55 3.54 3.53 3.53 3.52 3.52 3.51 3.51 3.50 3.44 3.43 3.41 3.41 3.39 3.38 3.37 3.26 3.24 3.23 3.21 2.52 DMSO 2.51 DMSO 2.51 DMSO 2.50 DMSO 2.50 DMSO 2.02
N
N
O
O
N
H
O
O
O
N3
4
0.74 0.89 1.89 2.54 9.59 2.35 3.03 2.44 3.00
10 9 8 7 6 5 4 3 2 1 0
1H
B
173.26 167.67 167.37 156.84 146.94 87.03 70.28 70.24 70.16 70.07 69.73 69.39 50.47 48.37 46.64 41.33 40.57 DMSO 40.37 DMSO 40.16 DMSO 39.95 DMSO 39.74 DMSO 39.53 DMSO 39.32 DMSO 39.18 39.10 16.93
180 170 160 150 140 130 120 110 100 90 80 70 60 50 40 30 20 10 0
13C

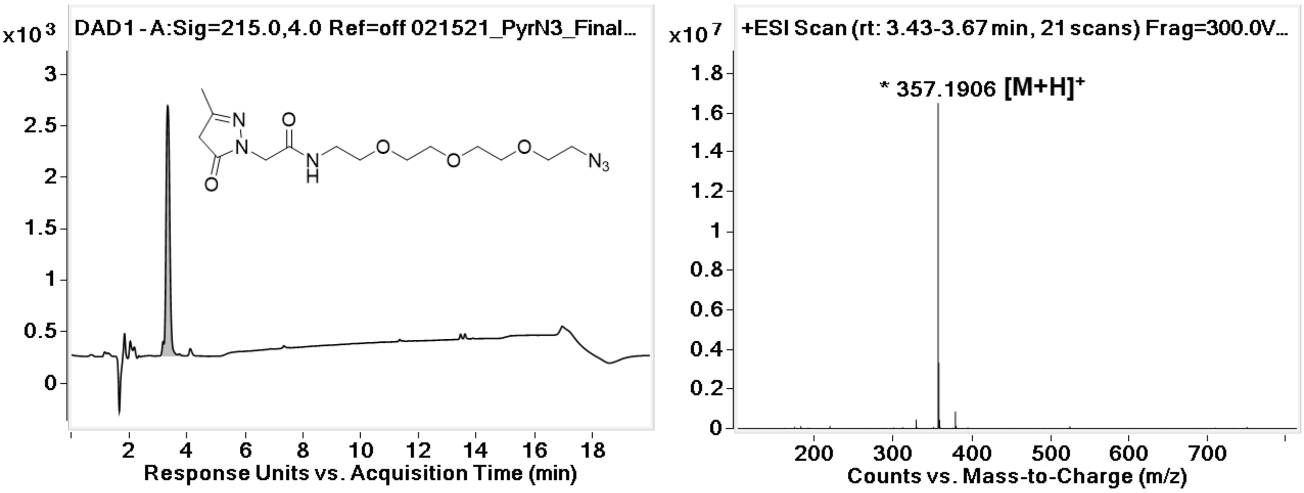
x10 3
DAD1 - A:Sig=215.0,4.0 Ref=off 021521_PyrN3_Final...
3
2.5
2
1.5
1
0.5
0
2 4 6 8 10 12 14 16 18
Response Units vs. Acquisition Time (min)
x10 7
+ESI Scan (rt: 3.43-3.67 min, 21 scans) Frag=300.0V...
* 357.1906 [M+H]+
1.8
1.6
1.4
1.2
1
0.8
0.6
0.4
0.2
0
200 300 400 500 600 700
Counts vs. Mass-to-Charge (m/z)

[1] A. T. Pedersen, W. R. Birmingham, G. Rehn, S. J. Charnock, N. J. Turner, J. M. Woodley, *Org. Process Res. Dev.* **2015**, *19*, 1580-1589.

[2] a) J. B. Rannes, A. Ioannou, S. C. Willies, G. Grogan, C. Behrens, S. L. Flitsch, N. J. Turner, *J. Am. Chem. Soc.* **2011**, *133*, 8436-8439; b) A. P. Mattey, W. R. Birmingham, P. Both, N. Kress, K. Huang, J. M. van Munster, G. S. Bulmer, F. Parmeggiani, J. Voglmeir, J. E. R. Martinez, N. J. Turner, S. L. Flitsch, *ACS Catal.* **2019**, *9*, 8208-8212.

[3] W. Duane Brown, *Biochim. Biophys. Acta* **1960**, *44*, 365-367.
